# T and NK Cells in IL2RG-Deficient Patient 50 Years After Hematopoietic Stem Cell Transplantation

**DOI:** 10.1007/s10875-022-01279-5

**Published:** 2022-05-09

**Authors:** Janine E. Melsen, Monique M. van Ostaijen-ten Dam, Erik B. van den Akker, Marij J. P. Welters, Kim C. Heezen, Ingrid Pico-Knijnenburg, P. Martijn Kolijn, Robbert G. M. Bredius, Remco van Doorn, Anton W. Langerak, Marco W. Schilham, Arjan C. Lankester

**Affiliations:** 1 Department of Pediatrics, Stem Cell Transplantation Program and Laboratory for Pediatric Immunology, Willem-Alexander Children’s Hospital, Leiden University Medical Center, Albinusdreef 2, 2333ZA Leiden, The Netherlands; 2 Department of Biomedical Data Sciences, Leiden University Medical Center, Leiden, The Netherlands; 3 Pattern Recognition & Bioinformatics, Delft University of Technology, Delft, The Netherlands; 4 Department of Medical Oncology, Leiden University Medical Center, Leiden, The Netherlands; 5 Department of Immunology, Erasmus Medical Center, Rotterdam, The Netherlands; 6 Department of Dermatology, Leiden University Medical Center, Leiden, The Netherlands

**Keywords:** Severe combined immune deficiency, hematopoietic stem cell transplantation, T cells, natural killer cells, T-cell receptor repertoire

## Abstract

The first successful European hematopoietic stem cell transplantation (HSCT) was performed in 1968 as treatment in a newborn with IL2RG deficiency using an HLA-identical sibling donor. Because of declining naive T and natural killer (NK) cells, and persistent human papilloma virus (HPV)-induced warts, the patient received a peripheral stem cell boost at the age of 37 years. NK and T cells were assessed before and up to 14 years after the boost by flow cytometry. The boost induced renewed reconstitution of functional NK cells that were 14 years later enriched for CD56^dim^CD27^+^ NK cells. T-cell phenotype and T-cell receptor (TCR) repertoire were simultaneously analyzed by including TCR Vβ antibodies in the cytometry panel. Naive T-cell numbers with a diverse TCR Vβ repertoire were increased by the boost. Before and after the boost, clonal expansions with a homogeneous TIGIT and PD-1 phenotype were identified in the CD27^−^ and/or CD28^−^ memory population in the patient, but not in the donor. TRB sequencing was applied on sorted T-cell subsets from blood and on T cells from skin biopsies. Abundant circulating CD8 memory clonotypes with a chronic virus-associated CD57^+^KLRG1^+^CX3CR1^+^ phenotype were also present in warts, but not in healthy skin of the patient, suggesting a link with HPV. In conclusion, we demonstrate in this IL2RG-deficient patient functional NK cells, a diverse and lasting naive T-cell compartment, supported by a stem cell boost, and an oligoclonal memory compartment half a century after HSCT.

**Supplementary Information:**

The online version contains supplementary material available at 10.1007/s10875-022-01279-5.

## Introduction

Hematopoietic stem cell transplantation (HSCT) is nowadays an established and curative treatment for hematological, immunological and malignant diseases. Identification of the human leukocyte antigen (HLA) system led to the concept of HLA matching and was key to the success of the first HSCT transplantations in 1968 [1, 2]. In that year, two patients with severe combined immunodeficiency (SCID) were successfully transplanted with bone marrow from their HLA-identical sibling, one in the USA and one in the Netherlands [3, 4]. The first European transplant recipient (hereafter named UPN1) suffered from SCID caused by a missense mutation in the X-linked IL2RG gene (c.184 T > C; p.Cys62Arg). This gene encodes the common γ chain of the receptor for IL-2 (IL2Rγ) and multiple other cytokines including IL-4, IL-7, IL-9, IL-15 and IL-21 [5]. Lack of a functional IL2Rγ results in the absence of T cells and natural killer (NK) cells, but the presence of B cells albeit with impaired function.

Reconstitution of an effective immune system after HSCT in IL2RG-deficient patients is of critical importance to combat infections. To monitor reconstitution of the NK and T cells, usually only the cell numbers are evaluated. However, the quality of the T cells in terms of T-cell receptor (TCR) repertoire is equally relevant [6]. The more diverse the TCR repertoire, the higher the chance that an antigen derived from an encountered pathogen is being recognized. Preferably, the TCR repertoire is simultaneously studied with the T-cell phenotype since maturation or differentiation of T cells is associated with skewing of the repertoire [7, 8]. However, the currently employed molecular techniques such as deep sequencing and spectratyping do not allow this simultaneous characterization unless the technique is applied at the single-cell level, which is costly and analytically more challenging.

To investigate the status of the T-cell compartment of UPN1 up to 51 years post-HSCT in the context of a stem cell boost at 37 years after HSCT, we aimed to combine detailed phenotyping with TCR repertoire analysis. Development of a T-cell-focused cytometry panel with a set of TCR Vβ antibodies allowed detailed exploration of the diversity, kinetics and phenotype of the T cells at the single-cell level. We identified a diverse naive T-cell repertoire and an oligoclonal memory T-cell compartment, which was confirmed by TRB sequencing. In addition, we zoomed in on the NK cells and demonstrated the expansion of a CD56^dim^CD27^+^ subset with low prevalence in controls. Altogether, we provide an in-depth immunological profile of the first European HSCT recipient with a unique follow-up.

## Methods

### Study Approval

With approval of the institutional review board (protocols P00.068, P01.028 and B17.001), blood (UPN1, healthy donor/controls) and skin samples (UPN1) were analyzed after informed consent was obtained from the hematopoietic stem cell transplant recipient (UPN1) and healthy donor/controls.

### Sample Processing

The peripheral blood mononuclear cells (PBMC) were isolated by Ficoll density gradient centrifugation and analyzed directly or after cryopreservation. Skin biopsies were directly cut into small pieces and further processed.

### NK Cell Stimulation

To study degranulation, PBMC were cocultured for 4 h with K562 cells. Anti-CD107a (Becton Dickinson (BD), FITC) was added to the PBMC at start of the coculture. Intracellular chemokine (CCL4, XCL1) and cytokine (IFN-γ) production was measured after stimulation with coated anti-CD16 for 4 h or K562 cells for overnight. For the CD16 stimulation, a flat-bottom plate was coated with 5 μg/ml goat anti-mouse antibody (BD) and subsequently with 1 μg/ml anti-CD16 (clone 3G8, Biolegend) in PBS. Mouse IgG1 isotype (1 μg/ml, Biolegend) served as negative control. Cells were cultured in AIM-V medium (Life Technologies, Bleiswijk, the Netherlands) supplemented with 10% FCS (Sigma–Aldrich, St. Louis, MO, USA) and 1% penicillin/streptomycin (Sigma–Aldrich) at 37 °C. For each cell culture condition, after 1 h of culture Golgistop (BD) was added.

### Conventional Flow Cytometry

PBMC were stained with fluorochrome-conjugated antibodies in PBS supplemented with 0.5% BSA and 2 mM EDTA, for 30 min at room temperature (RT). An eight-tube flow cytometry panel was designed to study the TCR Vβ expression and the T-cell phenotype. Each tube included three TCR Vβ antibodies from the IOTest Beta Mark Kit (Beckman Coulter (BC), Brea, CA, USA) and a backbone panel of the following surface markers: CD3, CD4, CD8, CCR7, CD45RA, CD27, CD28, TIGIT and PD-1. Details of the antibodies used are listed in Table S1A. DAPI (150 ng/ml, Sigma–Aldrich) was added prior to measurement to exclude dead cells. Data were acquired on a LSRII flow cytometer (BD) using FACS Diva software (v8.0, BD).

### Spectral Cytometry

PBMC were stained with fluorochrome-conjugated antibodies in PBS supplemented with Brilliant Stain buffer plus (BD), 0.5% BSA, 2 mM EDTA and 0.02% NaN3, for 30 min at RT. The TCR Vβ T-cell panel as described for conventional flow cytometry was supplemented with the following antibodies: CXCR6, CD127, CD57, TcRγδ, CD56, CX3CR1, CD16, NKG2A, CD95, DNAM1, KLRG1, CD69, CD103 and CLA (Table S1B). The NK phenotype panel included antibodies against CD69, DNAM1, CD57, CD3, CD45RA, CD62L, CD56, CD16, CX3CR1, CD7, KLRG1, KIRs, NKp46, CD127, NKG2A, NKG2D, TIGIT, NKG2C, CD45 and CD27 (Table S1B). The staining with unconjugated NKp46 antibody was performed prior to incubation with conjugated antibodies. PBMC were incubated with Fc block (eBioscience, San Diego, CA, USA) for 10 min at RT, followed by 30-min incubation with anti-NKp46, washing and 30-min incubation with secondary antibody. 7AAD or DAPI was added prior to acquisition to exclude dead cells. The peptide-stimulated PBMC were stained with Fixable Viability Dye eFluor 455UV to exclude dead cells prior to the extracellular staining. For the intracellular staining of stimulated PBMC, cells were fixed in 4% paraformaldehyde after extracellular staining and permeabilized in 0.1% saponin, as previously described [9]. Next, cells were incubated in Fc block for 10 min at RT. Intracellular staining with antibodies (Table S1B) was performed for 30 min at 4 °C. For the XCL1 staining, 6.7% donkey serum (Jackson ImmunoResearch, West Grove, PA, USA) was added together with unconjugated goat XCL1 to the mix. Finally, cells were incubated with secondary antibody to detect XCL1 for 30 min at 4 °C. Data were acquired on a 3L or 5L Aurora spectral cytometer (Cytek Biosciences, Fremont, CA, USA), using SpectroFlo software (v2.0, Cytek).

### Cell Sorting

PBMC from UPN1 and an age-matched healthy control were stained with fluorochrome-conjugated antibodies in PBS supplemented with 0.5% BSA and 2 mM EDTA, for 30 min at RT with the following antibodies: CD3, CD4, CD8, CCR7, CD45RA, CD27, CD28 and CD31 (Table S1C). Four populations were sorted per sample: naive CD4 and CD8, and CD27^−^ and/or CD28^−^ memory CD4 and CD8 T cells (Fig. S1). The populations were purified on an Aria I cell sorter (BD).

### Skin T-Cell Expansion

Skin biopsy pieces from wart enriched skin (wart^+^), developing warts (wart^±)^ and healthy skin (wart^−^) were incubated for 1 h at 37 °C in IMDM (Lonza, Breda, The Netherlands) supplemented with 10% human AB serum (Life Technologies), 50 µg/ml gentamycin (Life technologies), 25 µg/ml Fungizone (Life Technologies) and 10% penicillin/streptomycin. The skin pieces were cultured in IMDM supplemented with 7.5% human AB serum, 1% penicillin/streptomcyin and 1000 IU/ml IL-2 (Aldesleukin, Novartis, Arnhem, The Netherlands) for 26 days (culture medium). Half of the culture medium was refreshed once per two days with culture medium. When cells were overgrown, cells were divided over two or more wells in the culture medium.

### Peptide Stimulation

Thirteen 25-mer peptides with ten amino acids overlap (Table S2) from three distinct regions of the L1 protein of the HPV2 virus were synthesized (Peptide 2.0, Chantilly, VA, USA). Peptide stimulation of PBMC revealed two candidate immunogenic peptides. Next, PBMC were cultured with 1 μg/ml single peptides dissolved in DMSO or cultured with 0.002% DMSO only for 26 days. At day 6, brefeldin A (BFA, 1 μg/ml) was added to a fraction of cells for intracellular cytokine/chemokine detection at day 7. At day 13, cells were restimulated with autologous PBMC and 1 μg/ml peptide, and at day 20, 20 IU/ml IL-2 was added to the culture. Cells were harvested at day 26 for TRB sequencing. PBMC were cultured in AIM-V medium supplemented with 10% human AB serum and 1% penicillin/streptomycin at 37 °C.

### DNA Isolation

DNA was isolated from T cells cultured from skin biopsies, T cells sorted from blood, uncultured HPV-induced wart enriched skin tissue and PBMC stimulated with peptides. After resuspension in PBS, DNA from the cultured T cells was isolated using the genomic DNA isolation kit (Sigma–Aldrich), according to manufacturer’s instructions. The sorted blood T-cell subsets were washed in PBS and stored in RLT buffer plus supplemented with 1% β-mercaptoethanol at -80 °C. DNA was isolated from the lysates using the AllPrep DNA/RNA Mini kit (Qiagen, Hilden, Germany), according to manufacturer’s instructions. The HPV-induced wart enriched skin tissue was washed twice with PBS, resuspended in lysis buffer T supplemented with protease K (Sigma–Aldrich) and incubated for 3 h at 55 °C. RNAse and lysis buffer C were added, and the suspension was incubated for 45 min at 70 °C. After centrifugation, DNA from the supernatant was isolated by using the genomic DNA isolation kit according to manufacturer’s instructions. DNA from the peptide-stimulated PBMC was isolated using the QIAamp DNA microkit (Qiagen), according to manufacturer’s instructions. DNA was stored at -80 °C until further processing.

### PCR and Sequencing

75–100 ng of DNA was amplified through multiplex PCR of TRBV–TRBD–TRBJ gene rearrangements following the BIOMED-2 protocol [10]. Purification of PCR products and library preparation were performed as described previously [11, 12]. Paired-end sequencing was performed using the MiSeq Reagent Kit v2 (2 × 250 bp) on the MiSeq Benchtop Sequencer (Illumina, San Diego, CA, USA). To increase library diversity, PhiX was spiked-in at a 20% concentration. Raw FASTQ files were uploaded to the interactive ARResT/Interrogate immunoprofiler[13] for annotation of the reads and data exploration. QC metrics for the sequencing data are included in Table S3. A mean of 80,607 high-quality sequences was retrieved per sample. Unproductive sequences were removed from further analyses. Additional analyses were performed in R (version 3.5.0, R Foundation for Statistical Computing, Vienna, Austria). For determination of overlap of clonotypes among samples, clonotypes below 50 reads (potential sequencing artifacts) were removed. Shannon’s diversity was calculated using the package Abdiv [14]. The VDJdb database[15] was used, to study the presence of sequences with known antigen specificity. A matching clonotype was defined based on V, CDR3, J, and HLA-type restriction. Although the HLA type of the age-matched healthy control was unknown, the clonotypes of the healthy control that were matching with HLA-A-restricted clonotypes specific for EBV and CMV in the VDJdb database were all restricted to HLA-A*02:01.

### Cytometry Data Analysis

Kaluza (v2.1, BC), the OMIQ data science platform (Omiq, Inc., Santa Clara, CA, USA) and R were used for post-acquisition analysis. First, dead cells were excluded and single lymphocytes and monocytes were gated based on forward and side scatter. For spectral cytometry data, unwanted anomalies, based on changes in flow rate and outlier events, were removed by FlowAi [16]. NK cells were further selected as CD3^−^CD56^+^CD45RA^+^ cells. For conventional flow cytometry data, T cells were selected as CD3^+^, and three distinct TCR Vβ^+^ populations and 1 TCR Vβ^−^ population per individual tube were gated and exported as CSV in Kaluza (Figs. S2, S3). Parameters were Arcsinh-transformed (FSC-A and SSC-A were normalized to a 0–5 scale) and exported as FCS, using the FlowCore[17] package in R. The R code is available on GitHub (https://github.com/janinemelsen/Single-cell-analysis-flow-cytometry) [18]. Individual TCR Vβ populations were proportionally downsampled among tubes to include an equal number of T cells per tube per sample (Table S4). In total, 128 FCS files (4 samples × 8 tubes × 4 populations) were uploaded on the OMIQ platform. The CD4^−^CD3^high^ T cells were excluded from further analysis, since those T cells are likely to represent the γδ T cells. CD4^+^ and CD4^−^CD8^±^ were subsampled from each file (Fig. S3) and used as input for opt-SNE [19]. Opt-SNE was performed with default parameters (1000 iterations, 5000 opt-SNE end, 30 perplexity, theta 0.5). Clustering was performed with FlowSOM [20]. The opt-SNE embedding and the FlowSOM clustering were based on the forward scatter, side scatter, and the backbone panel of markers, excluding the TCR Vβ and CD3 expression (Fig. S2). Since the eight tubes were equally distributed among the clusters, we assumed that differences among the tubes were not driving the clustering (Fig. S4). For spectral cytometry data, the complete analysis was performed by using OMIQ, using the same approach except that the γδ parameter was used to exclude the γδ T cells. The inverse Simpson index[21] was calculated by using the package Abdiv [14].

### Statistics

Statistics were calculated in GraphPad Prism software (v9.0.1, GraphPad, La Jolla, CA, USA) by applying a one-way repeated measures ANOVA test. P-values below 0.05 were considered as statistically significant.

## Results

### Peripheral Stem Cell Boost Post-HSCT Induced Increased Donor Chimerism, NK Cell Reconstitution and Thymic Output in UPN1

**Fig. 1:**
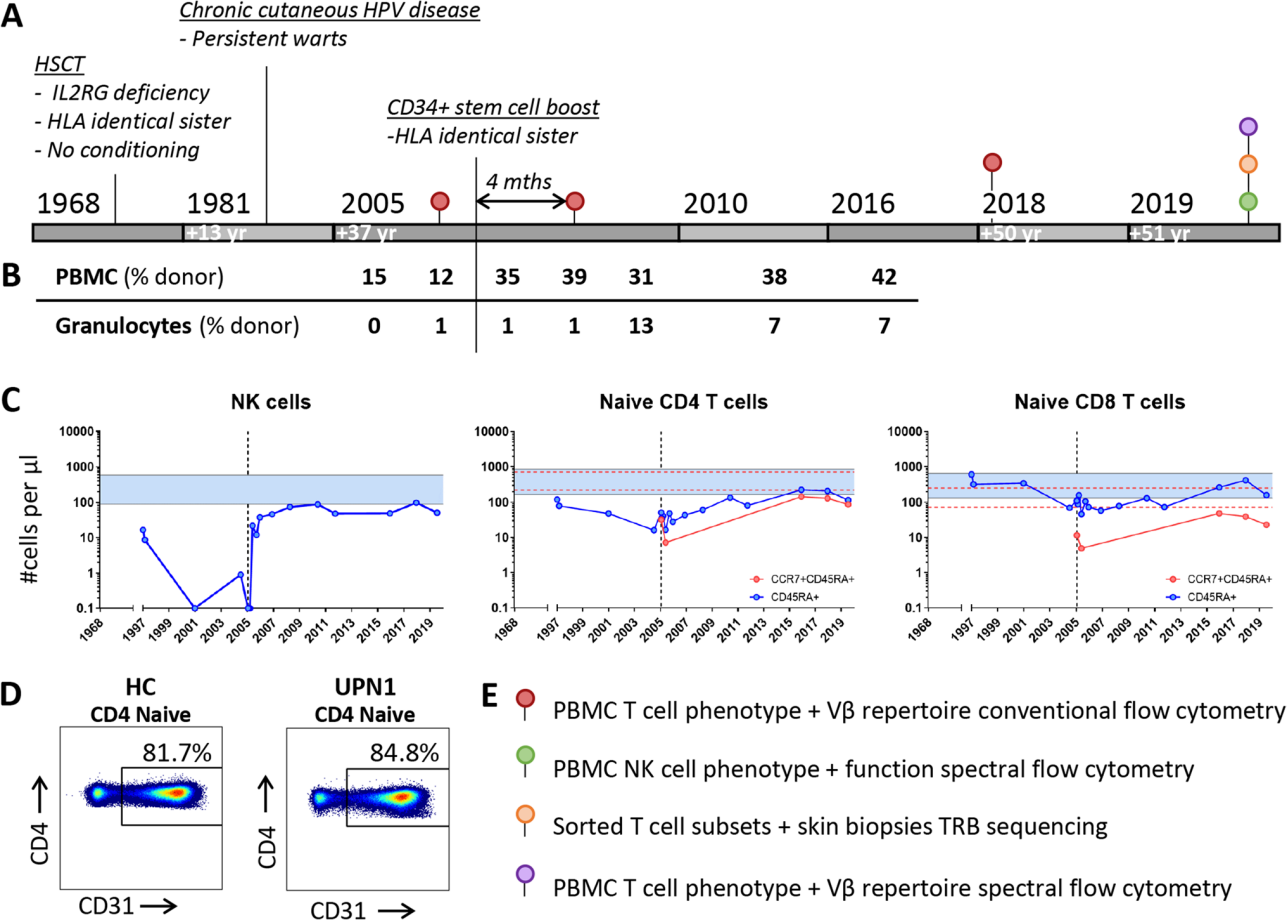
Clinical overview UPN1 and study design. **A**) Timeline indicates the hematopoietic stem cell transplantation (HSCT) in 1968, the onset of HPV disease and the CD34^+^-enriched peripheral stem cell boost in 2005. **B**) The donor chimerism of PBMC and granulocytes at different timepoints. **C**) The reconstitution of different lymphocyte subsets following HSCT and the boost (vertical dotted line). Naive T cells were either defined as CD45RA^+^ (blue) or CCR7^+^CD45RA^+^ (red). The horizontal lines represent the reference range of adult healthy donors. **D**) The CD31 expression on naive (CCR7^+^CD45RA^+^) CD4 T cells (surrogate marker of CD4 recent thymic emigrants) of an age-matched healthy control and UPN1 (51 years post-HSCT), as determined by flow cytometry. **E**) In this study, we assessed NK cell phenotype and function by spectral cytometry, and T-cell phenotype and repertoire by cytometry and TRB sequencing. The samples of UPN1 used for these analyses are indicated in A.

At the age of 22 weeks, UPN1 received a hematopoietic stem cell transplantation (HSCT) from his HLA-identical 7-year-old sister, without being conditioned (Fig. 1A). Since early adolescence the patient presented with lower limb lymphedema combined with recurrent skin infections, general malaise, and slowly progressive cutaneous warts mainly affecting the hands caused by human papilloma virus 2 (HPV2). At adult age, the latter appeared refractory to therapeutic interventions (including interferon treatment and surgical removal). Despite engraftment of the transplant, as demonstrated by 12–15% donor chimerism in the peripheral blood mononuclear cells (PBMC) (Fig. 1B), a gradual decline of natural killer (NK) cells and CD45RA^+^ CD4 and CD45RA^+^ CD8 T cells was observed (Fig. 1C). In addition, T-cell receptor excision circle (TREC) content at 32 years post-HSCT, as previously assessed by TREC analysis, was significantly lower in CD4 T cells (0.16 TRECs per µl blood) and CD8 T cells (0.02 TRECs per µl of blood) compared with his donor (39 yr, CD4 6.29 TRECs and CD8 4.87 TRECs per µl blood) [22]. For these reasons, UPN1 received a peripheral stem cell boost (CD34^+^ positively selected cells by CliniMACS®, 6.8*10^6^ CD34^+^/kg and 0.3*10^5^ CD3^+^/kg) from the same donor without prior conditioning, 37 years after HSCT. No graft versus host disease occurred. Following the boost, an increase in donor chimerism in both the PBMC (from 12% in 2005 to 42% in 2016) and granulocytes (from 1% in 2005 to 7% in 2016) was observed, which is in line with donor stem cell engraftment in the bone marrow (Fig. 1B). Moreover, the NK cells, which were absent prior to the boost, reconstituted after the boost (Fig. 1C). The total CD4 and CD8 T-cell numbers remained stable over 50 years, while the naive CD4 and CD8 T cells increased after the boost (Fig. 1C, S5A). At the age of 51, the CD31 expression on CD4 naive T cells, a hallmark for CD4^+^ recent thymic emigrants[23], was 84.8% compared to 81.7% in an age-matched healthy control (both values are above normal range[24]), indicative of a normal thymic output (Fig. 1D). The B cells steadily increased toward normal levels following the boost, while immunoglobulin levels remained unaffected, in the continued absence of immunoglobulin replacement therapy (Fig. S5A-B). The B-cell donor chimerism of 29%, 49% and 93% in the naive, unswitched memory and switched memory B cells, respectively, after the boost, indicates emergence of functional donor-derived B cells (Fig. S5C). The overall clinical condition significantly improved with less lymphedema and skin infections after the boost. Still, the HPV2 warts persisted although transient expansion and regression of the lesions were observed pointing to a certain level of HPV2 warts-directed immunity. Altogether, the clinical improvement and the increase in donor chimerism as well as the increasing NK cell and naive T-cell numbers after the boost underscore the effectiveness of the stem cell boost. To study in-depth the dynamics and longevity of the circulating NK cells and T cells isolated from blood and skin, we performed phenotypical, functional and repertoire analyses at multiple timepoints, up to 51 years post-HSCT (Fig. 1A,E).

### The Functional Circulating NK Cell Compartment in UPN1 is Enriched for CD56^dim^CD27^+^ NK Cells

**Fig. 2:**
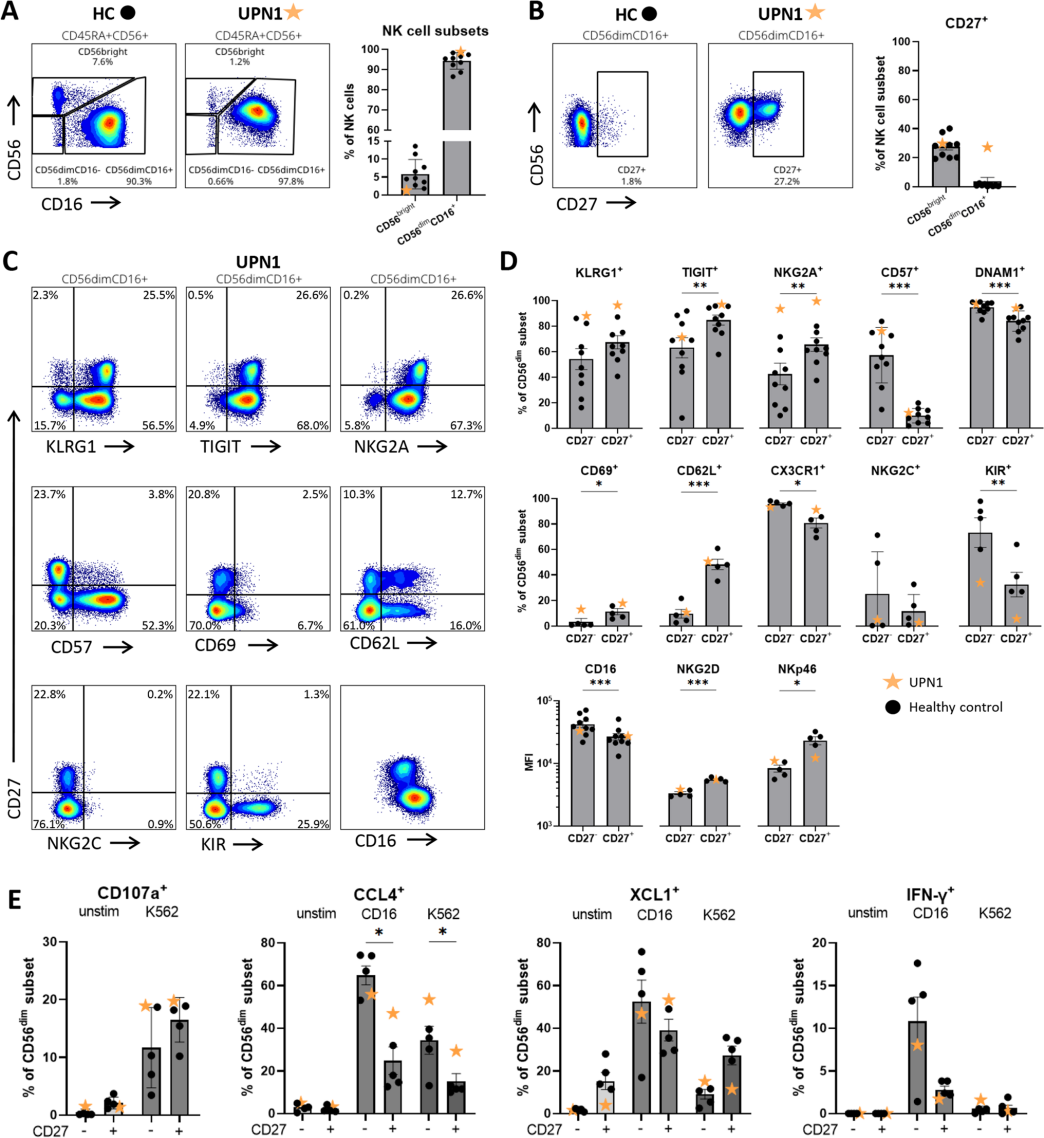
Enrichment of CD56^dim^CD27^+^ NK cells in functional circulating NK cell compartment. **A**) The density plots depict NK cells of a representative healthy control (HC) and UNP1 (14 years after the boost). The bar graphs indicate the frequency of CD56^bright^ and CD56^dim^CD16^+^ NK cells in blood of UPN1 (orange star) versus healthy controls (black dots, n = 9). **B**) The CD27 expression on the two NK cell subsets in healthy controls (n = 9) and UPN1. **C**) The CD56^dim^CD16^+^ NK cells were grouped into a CD27^−^ and CD27^+^ subset. The expression of multiple surface molecules on these subsets of UPN1 is depicted. **D**) Quantitative analysis of the expression of the surface molecules on NK cells of healthy controls (black dot, n = 4–9) versus UPN1 (orange star). **E**) NK cells from healthy controls (n = 4) and UPN1 were stimulated with K562 cells for 4 h to study degranulation (CD107a). Intracellular chemokine (CCL4, XCL1) and cytokine (IFN-γ) production was measured after stimulation by anti-CD16 (4 h) or K562 cells (overnight). Bar graphs in A, B, D and E indicate mean and standard error of the mean. One-way repeated measures ANOVA was applied to test for statistical differences between CD27^−^ and CD27^+^ NK cells. *p < 0.05, **p < 0.005, ***p < 0.001

While the initial HSCT did not lead to reconstitution of NK cells, the peripheral stem cell boost did induce NK cell reconstitution. However, we wondered whether the NK cells of UPN1 were phenotypically and functionally comparable to NK cells of healthy control. Fourteen years after the boost, a low frequency of CD56^bright^CD16^±^ NK cells was observed in UPN1 (1.2%) compared with healthy controls (median 6.2%, range 1.8–13.6%, Fig. 2A). Interestingly, CD27, a marker that is usually expressed by only a minor fraction of CD56^dim^CD16^+^ NK cells (mean 1.1% controls), was expressed by 27.2% of the CD56^dim^CD16^+^ NK cells of UPN1 (Fig. 2B). This CD56^dim^CD27^+^ subset was further characterized by higher expression of KLRG1, TIGIT, NKG2A, CD69, CD62L, NKG2D and NKp46 and lower expression of CD57, DNAM1, CX3CR1, KIRs and CD16, compared with the CD56^dim^CD27^−^ NK cells (Fig. 2C,D). The fact that cells with exactly this phenotype also exist in low numbers in healthy controls suggests that the CD56^dim^CD27^+^ NK cells are preferentially expanded in UPN1. To study the functional capacity of the NK cells, we measured degranulation, as well as the cytokine and chemokine response upon CD16 crosslinking and/or K562 stimulation. Although some differences in response were observed between the CD27^−^ and CD27^+^ CD56^dim^ NK cells, both subsets were capable of degranulation and production of CCL4, XCL1 and IFN-γ in all samples (Fig. 2E). In conclusion, the peripheral blood NK cells reconstituted by the boost were 14 years after the boost functional and enriched for a CD56^dim^CD27^+^ NK cell population.

### CD4 Subset Distribution Is Altered in UPN1 Compared with the Healthy Donor

**Fig. 3:**
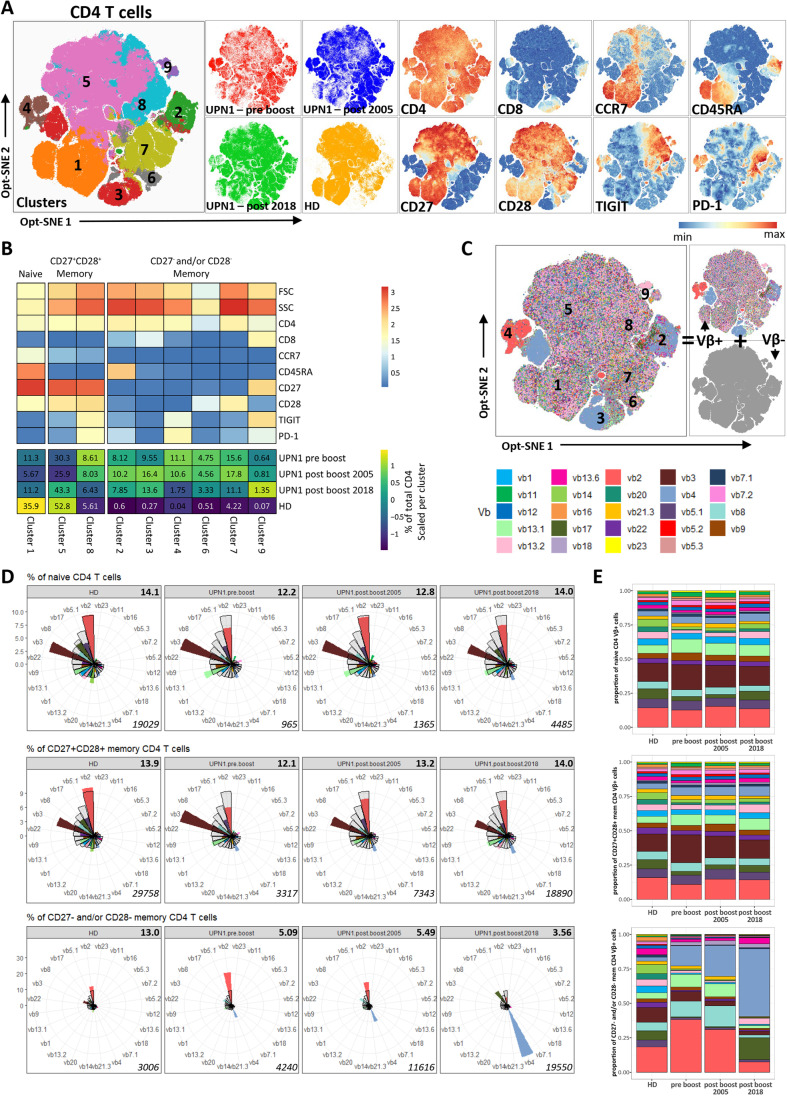
Clustering and Vβ frequencies of CD4 T cells. **A**) The clusters within the CD4 T-cell compartment, as determined by FlowSOM, are projected onto the opt-SNE embedding. The expression of each individual parameter is shown. No downsampling between samples was performed. **B**) The heatmap indicates the median expression value for each parameter per cluster. In addition, the frequencies of the different clusters in the four samples are depicted. **C**) After clustering, each cell was colored by its Vβ expression, which was previously determined by gating. **D**) Clusters were grouped into three groups: naive CD4 T cells (cluster 1), CD27^+^CD28^+^ memory CD4 T cells (clusters 5 and 8) and CD27^−^ and/or CD28^−^ memory CD4 T cells (clusters 2–4, 6, 7 and 9). The frequency of each individual Vβ was calculated as % of total T cells in the relevant tube and is shown per sample in a polar plot. The color indicates the frequency as measured within the sample, and the blanc bars indicate the mean reference values as determined among total CD4 T cells. The numbers at the right top and bottom represent the inverse Simpson index and total number of Vβ^+^ cells, respectively. **E**) The proportion of each individual Vβ among all Vβ^+^ cells is visualized per sample and subset.

Next, we studied the dynamics of the T-cell compartment in PBMC isolated prior to the boost, 4 months after the boost (2005), and 13 years after the boost (2018) by flow cytometry (Fig. S2). The panel consisted of eight tubes, each including markers specific for T-cell lineage (CD3, CD4, CD8), differentiation (CCR7, CD45RA, CD27, CD28), activation or exhaustion (TIGIT, PD-1) and receptor repertoire (3 TCR Vβ antibodies per tube, Fig. S2). As a control, PBMC isolated from the donor around the time of the boost, at the age of 40, were included. Within the blood CD4 T-cell compartment, nine clusters were assigned and visualized by opt-SNE (Fig. 3A). Based on the phenotype, clusters were subdivided into three groups: CCR7^+^CD45RA^+^CD27^+^CD28^+^ naive T cells (cluster 1), CD27^+^CD28^+^ early memory T cells (cluster 5,8) and CD27^−^ and/or CD28^−^ late memory T cells (cluster 2–4,6,7,9) (Fig. 3A,B). After the stem cell boost, there was a gradual increase of absolute numbers of naive T cells (Fig. 1C), while the frequency of naive T cells was not altered in the long term (11.3% pre-boost versus 11.2% post-boost 2018, Fig. 3B). In contrast, memory CD4 T cells that had lost CD27 and/or CD28 were nearly absent in the donor (5.7% of total CD4, sum of clusters 2–4, 6,7, 9), while they were abundant before and at the two time points after the stem cell boost in UPN1 (49.8%, 60.4%, 39.0% of total CD4, respectively, Fig. 3B).

### UPN1 Has a Diverse Naive CD4 TCR Vβ Repertoire Comparable to That of CD27^+^CD28^+^ Memory CD4 T Cells

After identifying the different subsets by clustering, we projected the TCR Vβ expression on the clusters and calculated the distribution of the TCR Vβs in the total CD4 T cells and within each cluster (Fig. 3C). Apart from an enrichment of TCR Vβ4 in UPN1, the TCR Vβ frequencies within the total CD4 T cells of the patient and donor were within normal or slightly below ranges of the reported reference values as measured in at least 46 healthy controls (Fig. S6A). When calculating the frequency of each TCR Vβ family within each CD4 T-cell subset, it became evident that in the naive and CD27^+^CD28^+^ early memory CD4 T cells, but not in the CD27^−^ and/or CD28^−^ memory population, each TCR Vβ family is present in UPN1 at a constant frequency over time and resembles the frequency in the donor (Fig. 3D). Strikingly, neither in the patient nor in the donor differences in the TCR Vβ family distribution were observed between the CD27^+^CD28^+^ memory and naive CD4 T cells (Fig. 3D). Therefore, the TCR Vβ repertoire of both the naive and CD27^+^CD28^+^ memory CD4 T cells is diverse and comparable to the healthy donor.

### Memory CD4 T Cells That Have Lost CD28 Are Enriched for Certain TCR Vβ Families and Cluster Based on a Consistent Phenotype of TIGIT and PD-1

**Fig. 4:**
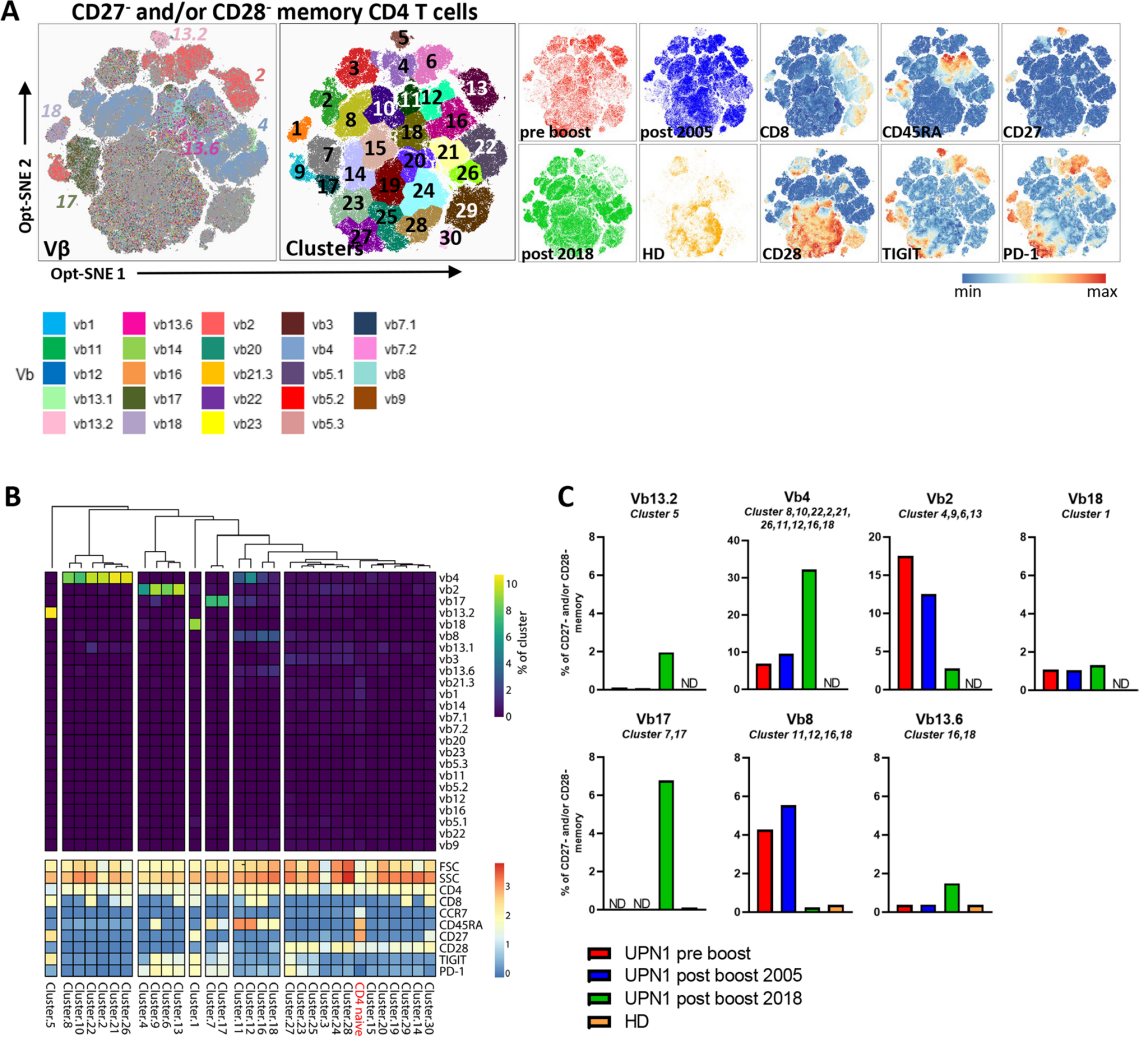
Skewed Vβ distribution within CD27^−^ and/or CD28^−^ memory CD4 T cells. **A**) An opt-SNE embedding is shown of CD27^−^ and/or CD28^−^ memory CD4 T cells with each cell colored by its Vβ expression, clustering, or parameter intensity. Clustering was based on opt-SNE coordinates. No downsampling was performed between samples. **B**) For each individual cluster, the Vβ frequency (of total cells in cluster) and the phenotype are visualized in a heatmap. From the UPN1 post-boost 2018 sample, the Vβ frequencies of the naive CD4 T-cell cluster as shown in Fig. 3A-C are added as reference. **C**) For each dominant Vβ family, the total frequency was calculated by the sum of the Vβ^+^ events of the indicated clusters divided by the sum of CD27^−^ and/or CD28^−^ memory CD4 T cells of the tube in which the particular Vβ antibody was included. ND = not detected

Within the memory CD4 T cells that lost CD27 and/or CD28, a highly restricted TCR Vβ repertoire was detected in the patient, as indicated by, for instance, the high frequency of TCR Vβ2 and Vβ4 and the low inverse Simpson index (Fig. 3D). In contrast, in the healthy donor the inverse Simpson index of these cells was not reduced compared with the naive cells (Fig. 3B,D). We therefore zoomed in on the CD27^−^ and/or CD28^−^ memory T cells, generated an opt-SNE and subclustered the data (Fig. 4A). Only one, two or three dominant TCR Vβs were observed within all the CD28^−^ subclusters from the patient, which is indicative of an oligoclonal expansion (Fig. 4A,B). To quantitatively determine which TCR Vβs are enriched, we calculated the frequency of each TCR Vβ within each cluster and compared the frequencies to the Vβ distribution of the naive CD4 T cells of the UPN1 post-boost 2018 sample (Fig. 3A-C). The clusters that did not cluster with the naive CD4 T cells in the heatmap were considered an oligoclonal expansion (Fig. 4B). For instance, in cluster 5, TCR Vβ13.2 was almost completely dominant. In this cluster, 10% of all the cells were represented by TCR Vβ13.2^+^ cells (the maximum is 12.5% since for each TCR Vβ^+^ cell, 7 Vβ^−^ cells from the other seven tubes are included in the cluster). Although CD4 memory T-cell differentiation is assumed to be associated with a loss of CD27, followed by a loss of CD28, we identified two CD28^−^ clusters (1 and 5) still expressing CD27 in which one TCR Vβ was dominant (Vβ18 and Vβ13.2, respectively). Notably, CD28^−^ CD4 T cells were barely present in the healthy donor (Fig. 3B, 4A), explaining the high diversity index of the CD27^−^ and/or CD28^−^ memory CD4 T cells. Together, these data indicate that the abundantly present CD28^−^ CD4 T cells have a skewed Vβ repertoire and are not derived from an adoptively transferred antigen-experienced CD28^−^ T-cell pool of the donor.

Interestingly, each cluster containing a dominant TCR Vβ family in UPN1 was characterized by a consistent expression pattern of TIGIT and PD-1. For instance, all the TCR Vβ13.2^+^ CD4 memory T cells in cluster 5 were positive for TIGIT and negative for PD-1, while the TCR Vβ4^+^ cells in clusters 8, 10, 22, 2, 21, 26, 11, 12, 16 and 18 expressed neither TIGIT nor PD-1 (Figs. 4A-B, S7). In contrast, some subclusters with TCR Vβ4 (11, 12, 16, 18), Vβ2 (9) and TCR Vβ17 (7, 17) dominance expressed a gradient of CD45RA, suggesting the presence of a differentiation gradient (Figs. 4A-B, S7). Similarly, a gradient of CD8 expression was observed on a fraction of all TCR Vβ dominant subclusters (5, 22, 21, 26, 13, 1, 12, 16), except for the TCR Vβ17^+^ family, ranging from 38% of TCR Vβ2^+^ cells to 92% of TCR Vβ13.2^+^ cells (Figs. 4A-B, S7). Therefore, it is important to consider CD4^+^CD8^dim^ T cells as conventional CD4 T cells, to prevent underestimation of the oligoclonal compartment.

Next, we evaluated the persistence of each TCR Vβ dominant expansion by determining the frequency in 2005 (pre-boost, 4 months post-boost) and 2018. The frequency of the clustered TCR Vβ8- and Vβ2-expressing cells was the highest in the pre- and post-boost 2005 samples and was decreased to a low level in 2018. The clustered TCR Vβ13.2^+^ and Vβ17^+^ cells were only present in the 2018 sample (Fig. 4C). The clustered TCR Vβ4^+^ cells represented more than 30% of the CD27^−^ and/or CD28^−^ memory CD4 T cells in 2018 and were present already in 2005 (Fig. 4C). The clustered TCR Vβ18^+^ and Vβ13.6^+^ cells were continuously detected at a level below 2% of the CD27^−^ and/or CD28^−^ memory CD4 T cells (Fig. 4C). Taken together, UPN1 has a dynamic T-cell compartment, characterized by CD28^−^ memory CD4 T-cell expansions, of which each has a dominant TCR Vβ expression and a unique surface marker phenotype.

### CD8 Compartment in UPN1 Is Characterized by Enrichment for CD27^−^ and/or CD28^−^ Memory T Cells

**Fig. 5:**
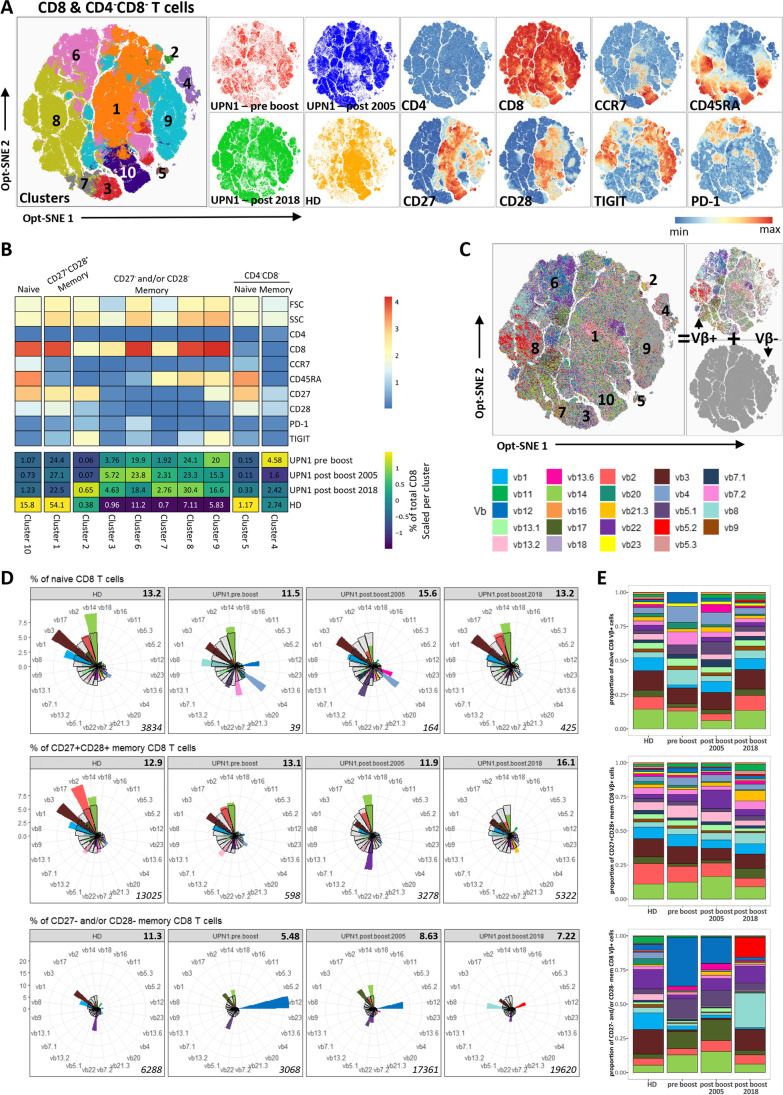
Clustering and Vβ frequencies of CD8 T cells. **A**) The clusters within the CD8 T-cell compartment, as determined by FlowSOM, are projected onto the opt-SNE embedding. The expression of each individual parameter is shown. No downsampling was performed between samples. **B**) The heatmap indicates the median expression value for each parameter per cluster. In addition, the frequencies of the different clusters in the four samples are depicted. **C**) After clustering, we colored each cell by its Vβ expression, which was previously determined by gating. **D**) Clusters were grouped into five groups: naive CD8 T cells (cluster 10), CD27^+^CD28^+^ memory CD8 T cells (cluster 1), CD27^−^ and/or CD28^−^ memory CD8 T cells (clusters 2,3,6,7,8,9), naive CD4^−^CD8^−^ T cells (cluster 5) and memory CD4^−^CD8^−^ T cells. A minor fraction of CD28^−^ cells in cluster 1 was manually removed. The frequency of each individual Vβ was calculated as % of total T cells in the relevant tube and is shown per sample in a polar plot. The color indicates the frequency as measured within the sample. The blanc bars indicate the mean reference values as determined among total CD8 T cells. The numbers at the right top and bottom represent the inverse Simpson index and total number of Vβ^+^ cells, respectively. **E**) The proportion of each individual Vβ among all Vβ^+^ cells is visualized per sample and subset.

To longitudinally study the CD8 and CD4^−^CD8^−^ compartment up to 50 years post-HSCT, we applied the same analysis approach and identified by clustering the naive CD8 T cells (cluster 10), CD27^+^CD28^+^ early memory CD8 T cells (cluster 1), CD27^−^ and/or CD28^−^ late memory CD8 T cells (cluster 2, 3, 6- 9), naive CD4^−^CD8^−^ T cells (cluster 5) and memory CD4^−^CD8^−^ T cells (cluster 4) (Fig. 5A,B). As for the CD4 T cells, the proportion of naive CD8 T cells was decreased, while the CD27^−^ and/or CD28^−^ memory CD8 T cells were enriched in UPN1 compared with his healthy donor. CD4^−^CD8^−^ T cells were present at a low frequency in both UPN1 and the healthy donor, varying from 1.7–4.7% (Fig. 5B).

### The Low Number of Naive CD8 T Cells Gives Rise to a Diverse CD27^+^CD28^+^ Memory Pool Prior to the Stem Cell Boost

Next, the TCR Vβ distribution was calculated among the total CD8 T cells (Fig. 5C). Except for the high frequency of TCR Vβ12 (18.3% sample 1) and Vβ5.2 (4.8% sample 3), the TCR Vβ frequencies of UPN1 and his donor were within the normal range for each Vβ (Fig. S6B). When zooming in on the naive CD8 T cells of UPN1, it became evident that the distribution of the TCR Vβs was solely comparable to the healthy donor 13 years after the stem cell boost, while the diversity index remained stable over time (Fig. 5D,E). Of note, the input naive CD8 TCR Vβ^+^ cells of the first two patient samples (39 and 164 cells) were fairly low compared with the naive CD4 TCR Vβ^+^ cells (965 and 1365 cells), probably resulting in a less accurate assessment of the TCR Vβ repertoire. The TCR Vβ distribution and diversity index of the CD27^+^CD28^+^ memory T cells were fairly similar between the donor and the patient in all samples, with the higher frequency of TCR Vβ22 in the UPN1 post-boost 2005 sample as exception (Fig. 5D,E). In the donor, the TCR Vβ distribution of the CD27^+^CD28^+^ memory T cells was reminiscent of the distribution of the naive T cells. Given the fact that the TCR Vβ distribution of the CD27^+^CD28^+^ memory T cells mirrors the naive T cells, as also shown in the CD4 population, we speculate that the TCR Vβ distribution of the naive CD8 T cells in the patient was probably before the boost already similar to the donor and therefore not altered by the boost.

### Multiple TCR Vβ Families Dominate the CD27^−^ and/or CD28^−^ Memory CD8 Compartment

**Fig. 6:**
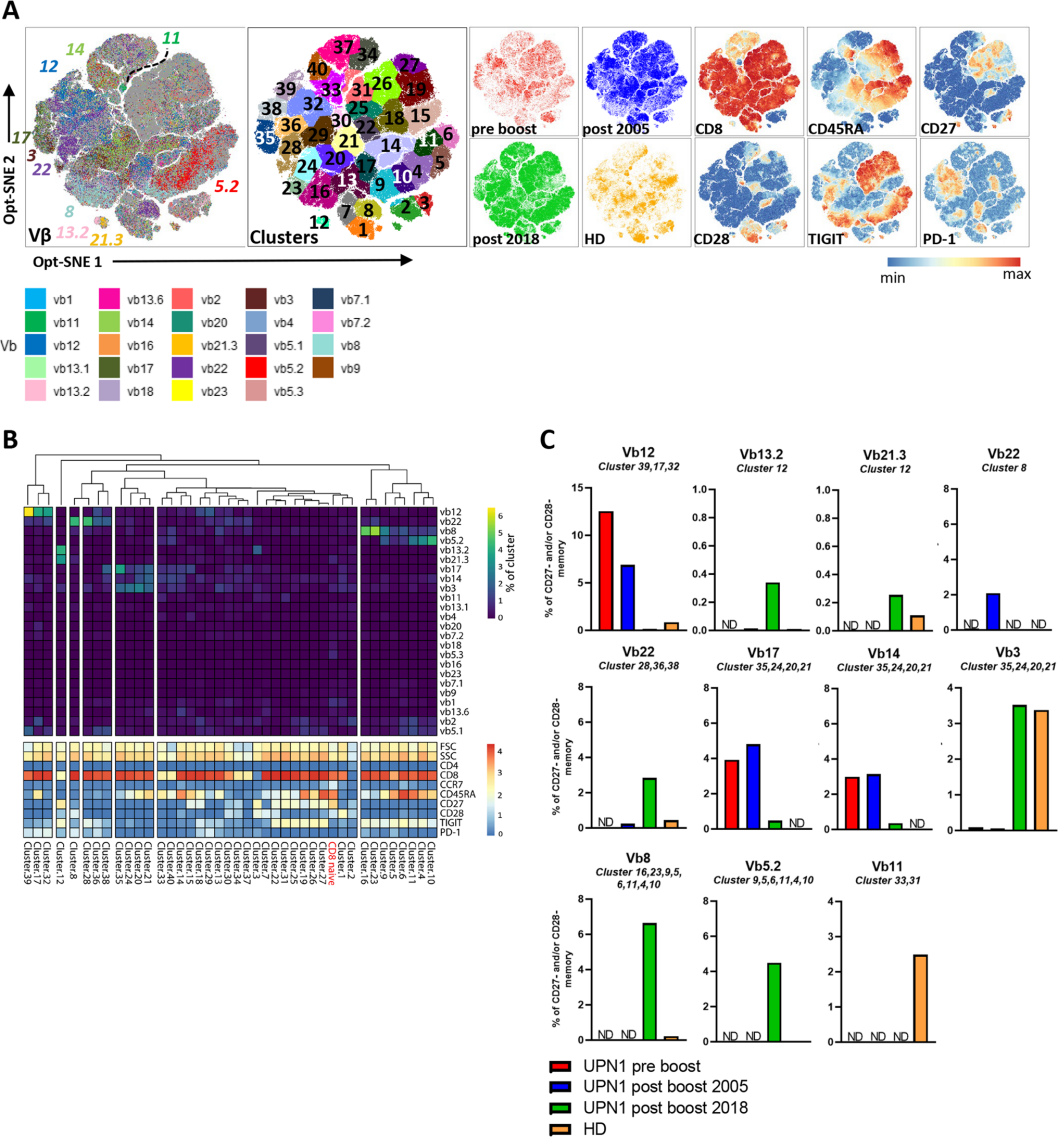
Skewed Vβ distribution within CD27^−^ and/or CD28^−^ memory CD8 T cells. **A**) An opt-SNE embedding is shown of CD27^−^ and/or CD28^−^ memory CD8 T cells with each cell colored by its Vβ expression, clustering, or parameter intensity. Clustering was based on opt-SNE coordinates. Between the samples, no downsampling was performed. **B**) For each individual cluster, the Vβ frequency (of total cells in cluster) and the phenotype are indicated in the heatmap. From the UPN1 post-boost 2018 sample, the Vβ frequencies of the naive CD8 T-cell cluster as shown in Fig. 5A-C are added as reference. **C**) For each dominant Vβ family, the total frequency was calculated by the sum of the Vβ^+^ events of the indicated clusters divided by the sum of CD27^−^ and/or CD28^−^ memory CD8 T cells of the tube in which the particular Vβ antibody was included. ND = not detected

The CD27^−^ and/or CD28^−^ memory CD8 T cells, analyzed separately, were characterized by a skewed TCR Vβ repertoire. The highest frequency observed was attributed to TCR Vβ12 in the pre-boost sample, which represented 22.3% of the CD27^−^ and/or CD28^−^ memory CD8 T cells (Fig. 5D). Half of the CD27^−^ and/or CD28^−^ memory CD8 T-cell clusters were considered an oligoclonal expansion, since they grouped separately from the naive CD8 T cells of the patient (Fig. 6B). In these clusters, multiple dominant TCR Vβ families were found, indicating an overlapping phenotype among clonally distinct T-cell populations (Fig. 6A,B). Each TCR Vβ family had a homogeneous phenotype concerning TIGIT and PD-1, while the CD45RA expression varied within one family. For instance, TCR Vβ12^+^ cells were enriched in CD45RA^−^ clusters 39, 32 and CD45RA^+^ cluster 17, but were all TIGIT^−^PD-1^+^ (Figs. 6B, S8). TCR Vβ8^+^ cells were enriched in CD45RA^dim^ clusters 16, 23 and CD45RA^high^ clusters 9, 5, 6, 11, 4 and 10, but overall had a TIGIT^+^PD-1^−^ phenotype. In contrast, within the TCR Vβ22^+^-enriched clusters two phenotypes were found, TIGIT^−^PD-1^high^ (cluster 8) and TIGIT^+^PD-1^low^ (cluster 28, 36, 38) (Fig. S8). This could indicate the presence of two clonally distinct CD8 T-cell expansions which express the same TCR Vβ. Within the CD4^−^CD8^−^ memory T cells (cluster 2, 3), a gradient expression of CD27 and CD28 was observed, and no dominant TCR Vβ families were identified.

When studying the kinetics, it became evident that also multiple dominant TCR Vβ families in the CD8 memory pool (Vβ12, Vβ17, Vβ14) persisted for at least 13 years, albeit at different frequencies Fig. 6C). One expansion was potentially transferred with the boost, since the clustered TCR Vβ3^+^ T cells were present in the donor at the same frequency as in the 2018 sample in the patient (3.5%, Fig. 6C). Notably, we found only one donor-specific dominant TCR Vβ family (Vβ11, part of cluster 31 and 33), representing 2.5% of CD27^−^ and/or CD28^−^ memory CD8 T cells (Fig. 6A,C). Taken together, the CD27^−^ and/or CD28^−^ memory CD8 T-cell population of UPN1 is characterized by the presence of multiple dominant TCR Vβ families, indicative of oligoclonal expansions.

### TRB Repertoire Sequencing Confirms Diversity of Naive and Oligoclonality of Memory Compartment in UPN1, Similar to an Age-Matched Healthy Control

**Fig. 7:**
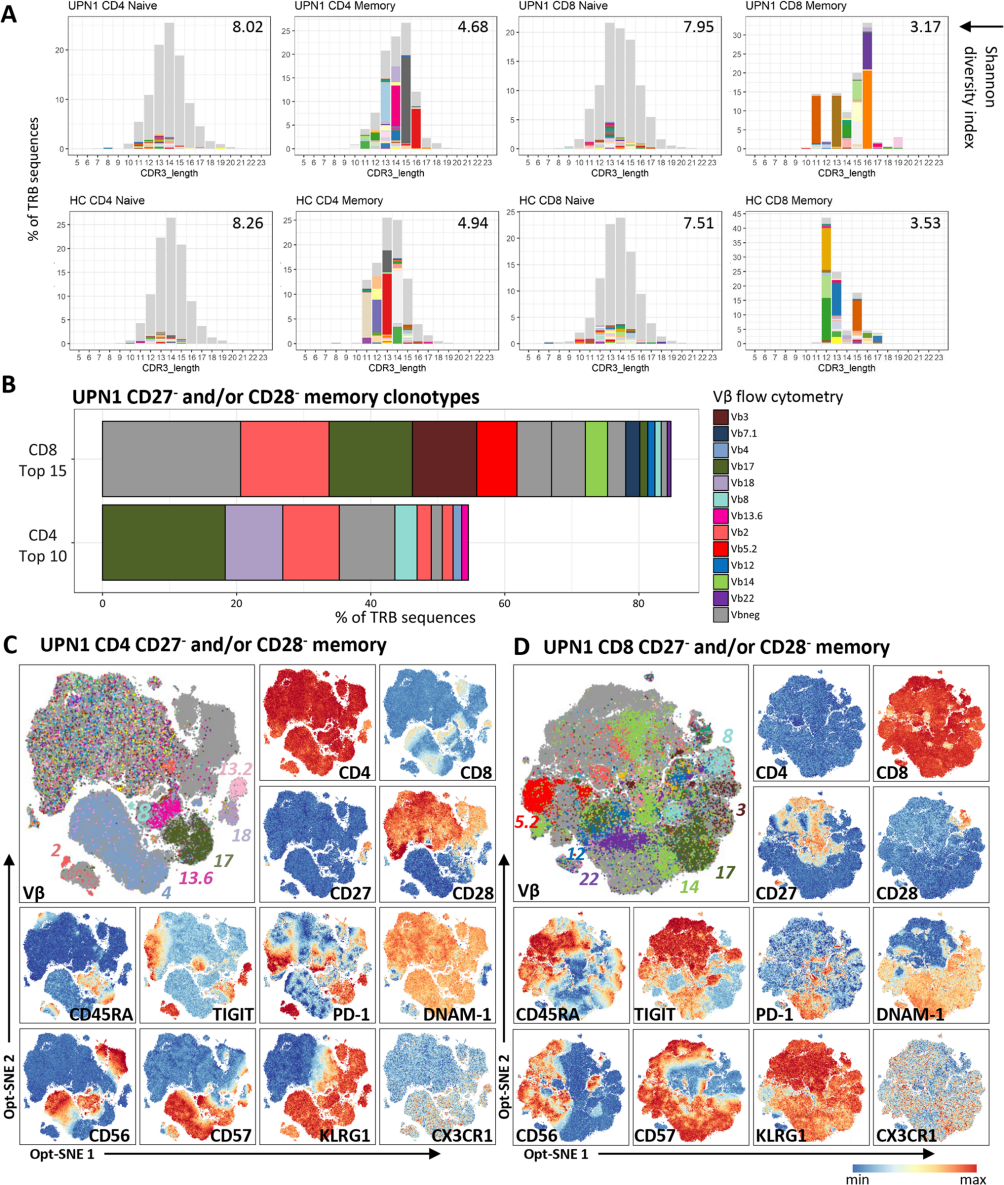
TRB sequencing of sorted blood T-cell subsets confirms clonality of expansions identified by flow cytometry. TRB sequencing of four peripheral blood T-cell subsets from UPN1 and an age-matched healthy control (HC) was performed: naive CD4, naive CD8, CD27^−^ and/or CD28^−^ memory CD4, and CD27^−^ and/or CD28^−^ memory CD8 T cells (Figure S1). **A**) The distribution of clonotypes based on CDR3 length is visualized with the top 50 most frequent clonotypes colored. The Shannon diversity index is shown at the right upper corner. **B**) The top ten most frequent clonotypes of the CD4 CD27^−^ and/or CD28^−^ memory T cells and the top 15 clonotypes of the CD8 CD27^−^ and/or CD28^−^ memory T cells are colored by their Vβ expression, with the same colors as applied for the flow cytometry analysis. Vbneg indicates a Vβ that was not detected by the antibodies included in the cytometry panel. **C**) Spectral cytometry was applied to provide a more detailed phenotype of the clonal expansions. Shown is an opt-SNE embedding based on the backbone panel of markers (except CD3) of the CD27^−^ and/or CD28^−^ memory CD4 T cells and **D**) the CD27^−^ and/or CD28^−^ memory CD8 T cells. The expression of Vβ and a selection of markers are shown.

To confirm our interpretations about the diversity of the naive T cells and to provide evidence that the cells expressing one of the dominant TCR Vβ families in the CD4 and CD8 CD27^−^ and/or CD28^−^ memory T-cell clusters are oligoclonal, we performed next-generation sequencing of the T-cell receptor β-chain. We sorted naive and CD27^−^ and/or CD28^−^ memory T-cell populations from blood (2019) from UPN1 and an age-matched healthy control (Fig. S1). Of note, in contrast to the donor of UPN1, the contribution of CD28^−^ cells to the CD27^−^ and/or CD28^−^ memory T cells of the age-matched control was comparable to UPN1 (Fig. S1A-C). The Shannon diversity score of the naive and memory T-cell populations was equal between UPN1 and the healthy control (Fig. 7A). The CDR3 length distribution plots with the top 50 most frequent clonotypes colored revealed that within the memory population of UPN1 and the healthy control some clonotypes represented 15–20% of all sequences (Fig. 7A). Altogether, these findings confirm that UPN1, similar to the healthy control, has a diverse naive and restricted memory CD4 and CD8 TCR repertoire.

### TRB Sequencing Confirms Clonality of Expansions Identified by Flow Cytometry

Six out of seven dominant TCR Vβ families (Vβ17, Vβ18, Vβ2, Vβ8, Vβ4 and Vβ13.6) previously identified by flow cytometry were included in the top ten most frequent clonotypes in CD4 memory T cells, confirming that the earlier discussed Vβ dominant clusters are mostly clonal T-cell expansions (Fig. 7B). In the CD8 memory T cells, seven of the nine dominant TCR Vβ families (Vβ17, Vβ3, Vβ5.2, Vβ14, Vβ12, Vβ8 and Vβ22) were matched to the top 15 clonotypes (Fig. 7B). We repeated the TCR Vβ analysis in a patient sample (2019), by using spectral cytometry to provide a more detailed phenotype of the clonal T-cell expansions. Seven dominant CD4 and seven dominant CD8 TCR Vβs were still present with the same TIGIT and PD-1 phenotype as earlier described (Fig. 7C, D). Multiple CD4 and CD8 clusters with only TCR Vβ’neg’ cells were identified, which is in line with the occurrence of clonotypes in the top 10 and top 15 that are not detected by the TCR Vβ antibodies present in the cytometry panel.

### Clonal CD4 and CD8 T-cell Expansions Have a CMV-Like Phenotype: CD57^+^KLRG1^+^CX3CR1^+^

Although UPN1 and his donor are seronegative for cytomegalovirus (CMV), the majority of the CD4 and CD8 clonal expansions in the patient expressed CD57, KLRG1 and CX3CR1, a phenotype which is associated with CMV infection (Fig. 7C, D). To validate this, we checked the CD4 and CD8 memory sequences of UPN1 and the age-matched healthy control in the VDJdb database which contains TCR sequences of known antigen specificity such as CMV and EBV. None of the clonotypes matched the clonotypes in the database, indicating that they are not associated with a currently known antigen specificity. In contrast, in the healthy control multiple memory clonotypes matched clonotypes specific for CMV and EBV, explaining the abundance of CD27^−^ and/or CD28^−^ memory T cells (Fig. S1, data not shown).

### Highly Frequent CD8 Memory Clonotypes Found in Blood Are Present in HPV2-Induced Warts

**Fig. 8:**
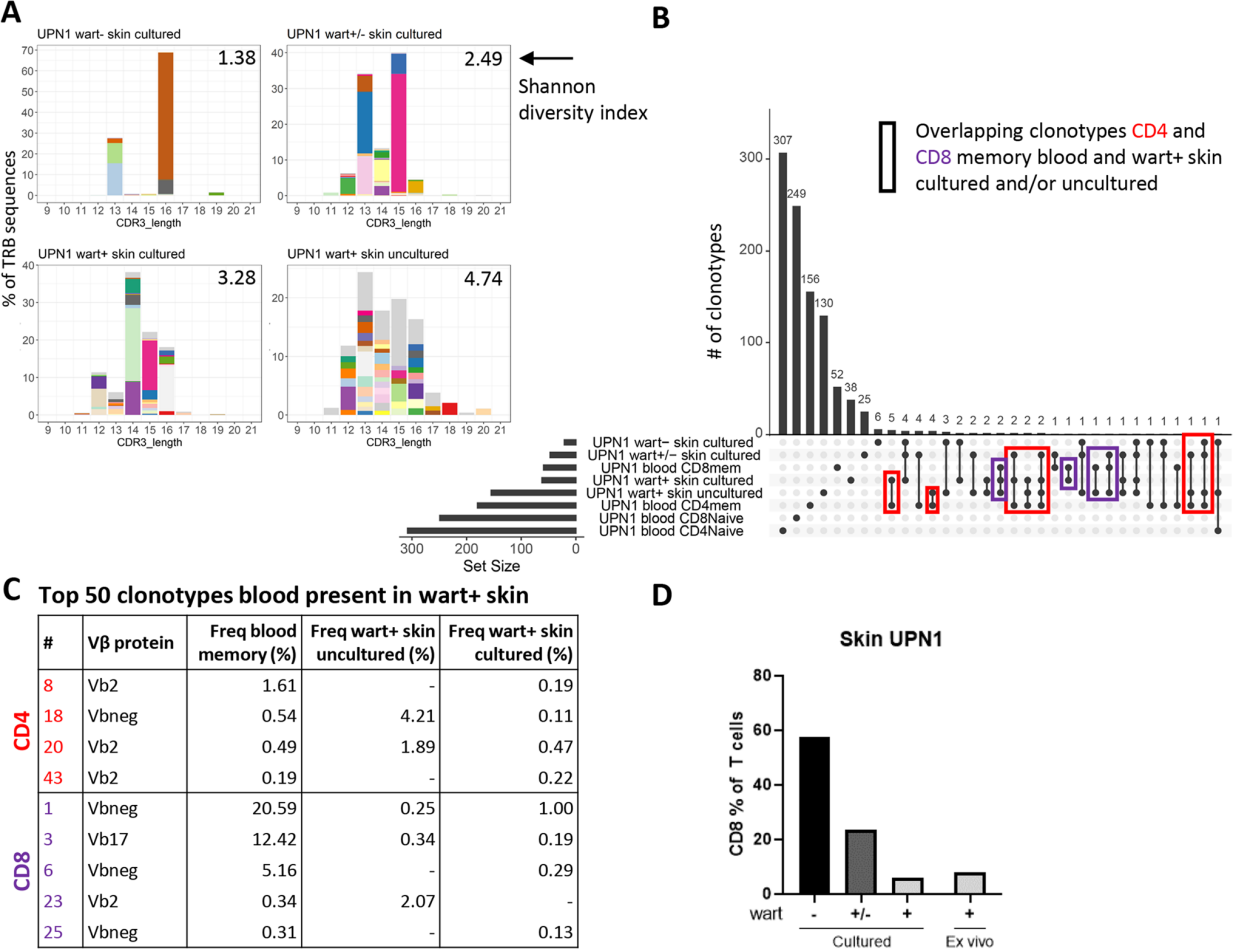
Abundant CD8 memory clonotypes in blood are present in HPV2-induced warts. **A**) TRB sequencing was performed on total T cells isolated from warts or total T cells isolated and expanded from wart^+^ skin, wart^±^ skin, or wart^−^ skin of UPN1. The frequencies of clonotypes based on CDR3 length are shown. The top 50 most frequent clonotypes are colored. The Shannon diversity index is shown at the right upper corner. **B**) The overlap of clonotypes among samples is shown. Only clonotypes with at least 50 reads were included. We focused on the clonotypes present in both CD4 memory (CD27^−^ and/or CD28^−^) from blood and uncultured or cultured T cells from wart^+^ skin (indicated by red, in total 17). The same strategy was applied for the memory CD27^−^ and/or CD28^−^ CD8 T cells (indicated in purple, in total 5). **C**) Of those overlapping sequences, we selected the ones present in the top 50 of most frequent clonotypes in the CD4 or CD8 memory compartment. The number indicates the ranking in the top 50. Vbneg indicates a Vβ that was not detected by the antibodies included in the cytometry panel. **D**) The percentage of CD8^+^ T cells in the skin samples of UPN1.

Since the clonotypes of UPN1 could not be linked to CMV, we wondered whether another chronic virus caused the phenotypic imprinting on the CD4 and CD8 memory T-cell compartment of the patient. Since the patient suffers from chronic HPV2-induced cutaneous warts, we aimed to study the link with HPV. First, the T-cell receptor β-chains of cultured T cells from wart^+^, wart^±^ and wart^−^ skin and of uncultured T cells from wart^+^ skin were sequenced. Overall, a skewed repertoire was found, also in the uncultured T cells of which the diversity index was comparable to the CD27^−^ and/or CD28^−^ memory CD4 T cells (Figs. 7A, 8A). We reasoned that if the observed expansions of clonotypes in blood would be related to recognition of HPV, there should be an overlap in TRB sequences between blood and affected skin. The overlap in sequences between blood and skin revealed that a few clonotypes observed in the memory pool were present in wart^+^ skin (Fig. 8B). In total, 17 clonotypes of the 180 clonotypes (with a cut-off value of 50 reads) of the CD4 CD27^−^ and/or CD28^−^ memory T cells in blood were observed in cultured and/or uncultured T cells from wart^+^ skin (Fig. 8B, red rectangles). Four out of these 17 were included in the top 50 most frequent CD4 clonotypes in blood; however, none of them matched the major clonal expansions observed by flow cytometry (Figs. 7C, 8C). In contrast, all the five overlapping CD8 memory clonotypes were present in the top 50 clonotypes of the blood memory CD8 T cells, of which one was detected by flow cytometry as well (TCR Vβ17, Fig. 8B, purple rectangles, 8C, 7D). The most frequent CD8 clonotype (representing 20.6% in blood) might match one of the CD27^−^CD28^−^ clusters, not recognized by any of the TCR Vβ antibodies in the panel (Figs. 7D, 8C). The frequency of the overlapping CD8 clonotypes in wart^+^ skin tissue was fairly low, ranging from 0.13% to 2.07% of all sequences. In line with this, the CD8 T cells, usually representing the vast minority of T cells in healthy skin[25], also had a low frequency in cultured (5.9%) and uncultured T cells (8.0%) from wart^+^ skin (Fig. 8D). To study whether the clonal expansions are specific for HPV2, we cultured PBMC from UPN1 in the presence of single peptides derived from the L1 protein of HPV2 (Table S2). After 7 days of stimulation, two peptides induced CCL4 and TNF-α production in CD8 T cells, suggesting the presence of HPV2-directed immunity (Fig. S9A). However, by TRB sequencing after an additional 19 days of culture, we did not observe expansion of the highly frequent clonotypes observed in blood (Fig. S9B). Thus, although we could not confirm the specificity of the major clonal expansions using our selection of HPV2-derived peptides, the presence of clonotypes from the circulating CD8 CD27^−^ and/or CD28^−^ memory pool in HPV-induced warts suggests a link with HPV.

## Discussion

In this study, we focused on the diversity and dynamics of the T- and NK cell compartment of an IL2RG-deficient patient who received an unconditioned HSCT more than 50 years ago and a stem cell boost 14 years ago. At the age of 51 years, an enrichment of CD56^dim^CD27^+^ NK cells was observed, with normal effector function compared with healthy controls. Moreover, the size and the TRB diversity of the naive T-cell compartment were comparable to an age-matched healthy control. Simultaneous characterization of the T-cell phenotype and TCR Vβ repertoire with a single-cell analysis approach led to the identification of clonal memory T-cell expansions with distinct differentiation stages, confirmed by TRB sequencing. The highly frequent CD8 memory clonotypes were also present in HPV-induced warts, suggesting a link with the persistent HPV infection in the patient.

HSCT in the absence of a pretransplant conditioning regimen for IL2RG deficiency is associated with low donor chimerism in the myeloid compartment and poor NK cell engraftment [26–28]. This was also observed for UPN1, together with a shortage and decline of naive T cells. To increase thymic output, he received an unconditioned peripheral CD34^+^-enriched stem cell boost 37 years post-HSCT. The fact that donor chimerism increased in the myeloid cells, that NK cells reconstituted, and that naive T-cell numbers increased following the boost points to a successful engraftment of donor hematopoietic stem cells, despite the lack of conditioning. Although the absolute number of naive T cells was very low prior to the boost, the repertoire based on the TCR Vβ distribution in the naive CD4 compartment was still comparable to his healthy donor. The low number of naive CD8 T cells included in our analysis prior to the boost was too low to draw firm conclusions. Still, the TCR Vβ diversity of the CD27^+^CD28^+^ memory CD8 T cells indicated that the low number of naive CD8 T cells was capable of giving rise to a diverse CD27^+^CD28^+^ memory compartment prior to the boost. Because of the pre-existing normal TCR Vβ diversity in the naive and early memory compartment, the stem cell boost induced mainly a quantitative and likely less of a qualitative increase in naive T cells. In line with the literature, 14 years after the boost, the thymus still supported T-cell development, as evidenced by the high percentage of CD4 recent thymic emigrants and presence of naive T cells with a high TRB diversity [29].

When analyzing the TCR repertoire, it is important to consider the different T-cell subsets, based on differentiation status, rather than the T-cell compartment as a whole. Our multi-parametric single-cell analysis approach led to the identification of minor and major clonal expansions in the CD27^−^ and/or CD28^−^ memory T-cell population, of which most were not detected by conventional analysis. Interestingly, each clonal expansion was characterized by a homogeneous phenotype of TIGIT and PD-1 that remained stable over the years. This suggests that the repertoire of checkpoint molecules might have been imprinted at an earlier stage of the T-cell response. Taking into account the relatively short life span of less than 6 months that has been reported in the literature, this raises the question how these memory T-cell expansions are maintained in this patient for the long period of at least 14 years [30–32]. Whether a clonal population is replenished by naive or stem cell memory T cells, or maintained by self-renewal is still under debate [30, 33, 34].

The CD57^+^KLRG1^+^CX3CR1^+^ phenotype of both the CD4 and CD8 clonal expansions is indicative of a late effector cytotoxic stage and is usually associated with chronic CMV infection [35–38]. Since the patient is CMV seronegative and no clonotypes known to be specific for CMV were detected, another chronic virus infection might be responsible for this phenotypical footprint on the memory compartment. From his teenage years, the patient suffered from chronic cutaneous lesions caused by HPV2. Although we could not confirm HPV specificity of the CD27^−^ and/or CD28^−^ memory T cells by peptide stimulations, we did find overlapping clonotypes between blood and skin lesions. Interestingly, chronic severe HPV infection after HSCT has been specifically reported in patients deficient for IL2RG, or its signaling partner JAK3, suggesting a role for the underlying genetic defect in other cells than the ones replaced by the transplantation [27, 39]. One hypothesis attributes the intrinsic defect of keratinocytes to the lack of HPV clearance. A keratinocyte cell line deficient for IL2RG showed a reduced chemokine response after IL-15 stimulation, resulting in reduced dendritic cell and CD4 T-cell migration [40]. This hypothesis is in agreement with the low frequency of overlapping CD8 clonotypes in wart^+^ skin. Similarly, the IL2RG deficiency in the host dendritic or Langerhans cells might result in inefficient T-cell activation, survival or migration. Yet, HPV disease also occurred in patients with full donor chimerism [39]. The other hypothesis suggests a role for the low prevalence or dysfunctionality of NK cells [41, 42]. However, in our patient normal NK cell numbers and functionality were observed after the boost without significant impact on HPV lesions. We speculate that the expanded CD56^dim^CD27^+^ NK cells represent an intermediary between the CD56^bright^ and major CD56^dim^CD27^−^ subset [43, 44]. Further studies are ongoing to examine whether this is related to HPV or other extrinsic factors. It is very well imaginable that in the heterogenous population of IL2RG patients, a combination of individual factors contributes to the chronic HPV infection.

In conclusion, we demonstrate that more than 50 years after unconditioned HSCT and 14 years after a stem cell boost in an IL2RG-deficient SCID patient, a diverse and lasting naive T-cell compartment has developed including persistent clonal T-cell expansions that might be linked to HPV. Further research will be required to determine whether our observations are patient or population specific.

## Supplementary Information

Below is the link to the electronic supplementary material.

Supplementary file1 (PDF 2099 KB)

## Data Availability

Raw data will be available upon request.
