# Supplementary material for: T and NK Cells in IL2RG-Deficient Patient 50 Years After Hematopoietic Stem Cell Transplantation
Source: J Clin Immunol. 2022 May 9;42(6):1205–22. doi: 10.1007/s10875-022-01279-5 (PMC9537207; doi:10.1007/s10875-022-01279-5)
Supplement: Supplementary file 1 — Supplementary file1 (PDF 2099 KB) [file 10875_2022_1279_MOESM1_ESM.pdf]

Table S1 Antibodies

A. Antibodies conventional flow cytometry

Specificity		Antibody characteristics				
**CD designation**	**Alternative name**	**Fluorochrome**	**Isotype**	**Clone**	**Company**	**Catalog#**
	Vb5.3	PE	mouse IgG1	3D11	Beckman Coulter	IM3497
	Vb7.1	PE+FITC	mouse IgG2a	ZOE	Beckman Coulter	IM3497
	Vb3	FITC	mouse IgM	CH92	Beckman Coulter	IM3497
	Vb9	PE	mouse IgG2a	FIN9	Beckman Coulter	IM3497
	Vb17	PE+FITC	mouse IgG1	E17.5F3	Beckman Coulter	IM3497
	Vb16	FITC	mouse IgG1	TAMAYA1.2	Beckman Coulter	IM3497
	Vb18	PE	mouse IgG1	BA62.6	Beckman Coulter	IM3497
	Vb5.1	PE+FITC	mouse IgG2a	IMMU157	Beckman Coulter	IM3497
	Vb20	FITC	mouse IgG2a	ELL1.4	Beckman Coulter	IM3497
	Vb13.1	PE	mouse IgG2b	IMMU222	Beckman Coulter	IM3497
	Vb13.6	PE+FITC	mouse IgG1	JU74.3	Beckman Coulter	IM3497
	Vb8	FITC	mouse IgG2a	56C5.2	Beckman Coulter	IM3497
	Vb5.2	PE	mouse IgG1	36213	Beckman Coulter	IM3497
	Vb20	PE+FITC	mouse IgG1	MPB2D5	Beckman Coulter	IM3497
	Vb12	FITC	mouse IgG2a	VER2.32	Beckman Coulter	IM3497
	Vb23	PE	mouse IgG1	AF23	Beckman Coulter	IM3497
	Vb1	PE+FITC	rat IgG1	BL37.2	Beckman Coulter	IM3497
	Vb21.3	FITC	mouse IgG2a	IG125	Beckman Coulter	IM3497
	Vb11	PE	mouse IgG2a	C21	Beckman Coulter	IM3497
	Vb22	PE+FITC	mouse IgG1	IMMU546	Beckman Coulter	IM3497
	Vb14	FITC	mouse IgG1	CAS1.1.3	Beckman Coulter	IM3497
	Vb13.2	PE	mouse IgG1	H132	Beckman Coulter	IM3497
	Vb4	PE+FITC	rat IgM	WJF24	Beckman Coulter	IM3497
	Vb7.2	FITC	mouse IgG2a	ZIZOU4	Beckman Coulter	IM3497
CD3	CD3	BV510	mouse IgG1	UCHT1	Biolegend	300448
CD4	CD4	AF700	mouse IgG1	SK3	Biolegend	344622
CD8	CD8	PE-Cy7	mouse IgG1	SK1	Becton Dickinson	335822
CD27	CD27	BV421	mouse IgG1	M-T271	Becton Dickinson	562513
CD28	CD28	PE-Cy5.5	mouse IgG1	CD28.2	Beckman Coulter	B24027
CD45RA	PTPRC	ECD	mouse IgG2b	MEM-56	Invitrogen	335039
CD197	CCR7	APC-Cy7	mouse IgG2a	G043H7	Biolegend	353212
CD297	PD-1	BV711	mouse IgG1	EH12.1	Becton Dickinson	564017
	TIGIT	APC	mouse IgG1	MBSA43	eBioscience	179500

B. Antibodies spectral cytometry

Specificity		Antibody characteristics				
**CD designation**	**Alternative name**	**Fluorochrome**	**Isotype**	**Clone**	**Company**	**Catalog#**
	Vb5.3	PE	mouse IgG1	3D11	Beckman Coulter	IM3497
	Vb7.1	PE+FITC	mouse IgG2a	ZOE	Beckman Coulter	IM3497
	Vb3	FITC	mouse IgM	CH92	Beckman Coulter	IM3497
	Vb9	PE	mouse IgG2a	FIN9	Beckman Coulter	IM3497
	Vb17	PE+FITC	mouse IgG1	E17.5F3	Beckman Coulter	IM3497
	Vb16	FITC	mouse IgG1	TAMAYA1.2	Beckman Coulter	IM3497
	Vb18	PE	mouse IgG1	BA62.6	Beckman Coulter	IM3497
	Vb5.1	PE+FITC	mouse IgG2a	IMMU157	Beckman Coulter	IM3497
	Vb20	FITC	mouse IgG2a	ELL1.4	Beckman Coulter	IM3497
	Vb13.1	PE	mouse IgG2b	IMMU222	Beckman Coulter	IM3497
	Vb13.6	PE+FITC	mouse IgG1	JU74.3	Beckman Coulter	IM3497
	Vb8	FITC	mouse IgG2a	56C5.2	Beckman Coulter	IM3497
	Vb5.2	PE	mouse IgG1	36213	Beckman Coulter	IM3497
	Vb20	PE+FITC	mouse IgG1	MPB2D5	Beckman Coulter	IM3497
	Vb12	FITC	mouse IgG2a	VER2.32	Beckman Coulter	IM3497
	Vb23	PE	mouse IgG1	AF23	Beckman Coulter	IM3497
	Vb1	PE+FITC	rat IgG1	BL37.2	Beckman Coulter	IM3497
	Vb21.3	FITC	mouse IgG2a	IG125	Beckman Coulter	IM3497
	Vb11	PE	mouse IgG2a	C21	Beckman Coulter	IM3497
	Vb22	PE+FITC	mouse IgG1	IMMU546	Beckman Coulter	IM3497
	Vb14	FITC	mouse IgG1	CAS1.1.3	Beckman Coulter	IM3497
	Vb13.2	PE	mouse IgG1	H132	Beckman Coulter	IM3497
	Vb4	PE+FITC	rat IgM	WJF24	Beckman Coulter	IM3497
	Vb7.2	FITC	mouse IgG2a	ZIZOU4	Beckman Coulter	IM3497
CD3		BV480	mouse IgG1	UCHT1	Becton Dickinson	566105
CD4		AF532	mouse IgG1	SK3	Thermofisher	58-0047-42
CD4		APC-H7	mouse IgG1	RPA-T4	Becton Dickinson	560158
CD7		AF532	mouse IgG1	eBio124-1D1	eBioscience	58-0079-42

CD8		AF700	mouse IgG1	SK1	Biolegend	344724
CD8		BV605	mouse IgG1	SK1	Becton Dickinson	564116
CD16	FCGR3A	BV711	mouse IgG1	3G8	Becton Dickinson	563127
CD16	FCGR3A	Vio515	mouse IgG1	REA423	Miltenyi	130-119-616
CD27	TNFRSF7	APC-Fire810	mouse IgG1	QA17A18	Biolegend	393214
CD27	TNFRSF7	BV605	mouse IgG1	L128	Becton Dickinson	562655
CD28		BV750	mouse IgG1	CD28.2	Becton Dickinson	747329
CD28		BUV661	mouse IgG1	CD28.2	Becton Dickinson	741635
CD45		APC-H7	mouse IgG1	2D1	Becton Dickinson	641417
CD45RA		BV570	mouse IgG2b	HI100	Biolegend	304132
CD56	NCAM	BV650	mouse IgG2b	NCAM16.2	Becton Dickinson	564057
CD159a	NKG2A	PE	mouse IgG2b	Z199	Beckman Coulter	IM3291U
CD103	ITGAE	FITC	mouse IgG1	BER-ACT8	DAKO	F7138
CD186	CXCR6	BV421	mouse IgG2a	K041E5	Biolegend	356014
CD127	IL7R	SB436	mouse IgG1	eBioRDR5	Thermofisher	62-1278-42
CD57	B3GAT1	PB	mouse IgM	HNK1	Biolegend	359608
CD62L	SELL	BV605	mouse IgG1	DREG-56	Biolegend	304834
CD69		BUV805	mouse IgG1	FN50	Biolegend	748763
CD69		PE-Cy7	mouse IgG1	FN50	Biolegend	310912
CD95	FAS	PE-Dazzle594	mouse IgG1	DX2	Biolegend	305634
CD107a	LAMP1	FITC	mouse IgG1	H4A3	Becton Dickinson	555800
CD127	IL7Ra	PE-Cy5.5	mouse IgG1	eBioRDR5	eBioscience	35-1278-42
CD158a+h	KIR2DL1, KIR2DS1	PE	mouse IgG1	EB6	Beckman Coulter	A09778
CD158b1+b2+j	KIR2DL2, KIR2DL3, KIR2DS2	PE	mouse IgG1	GL183	Beckman Coulter	IM2278U
CD158e1	KIR3DL1	PE	mouse IgG1	DX9	Becton Dickinson	555967
CD158i	KIR2DS4	PE	mouse IgG2a	FES172	Beckman Coulter	IM3337
CD159a	NKG2A	APC	mouse IgG2b	Z199	Beckman Coulter	A60797
CD159a	NKG2A	PE-Cy7	mouse IgG2b	Z199	Beckman Coulter	B10246
CD159c	NKG2C	AF700	mouse IgG1	134591	R&D Systems	FAB138N-100
CD197	CCR7	APC-Fire750	mouse IgG2a	G043H7	Biolegend	353246
CD226	DNAM-1	BUV563	mouse IgG1	DX11	Becton Dickinson	748429
CD226	DNAM-1	BB700	mouse IgG1	DX11	Becton Dickinson	745864
CD279	PD-1	BV711	mouse IgG1	EH12.1	Becton Dickinson	564017
CD314	NKG2D	APC	mouse IgG1	1D11	Becton Dickinson	558071
CD335	NKp46	unconjugated	mouse IgG1	9E2	BD	557911
	CCL4	PerCP-ef710	mouse IgG2a	FL34Z3L	eBioscience	46-7540-42
	CLA	APC	rat IgM	HECA-452	Miltenyi	130-098-573
	CX3CR1	BV785	mouse IgG2b	2A9-1	Becton Dickinson	744489
	IFN-γ	PE-Dazzle594	mouse IgG1	4S.B3	Biolegend	502546
	IFN-γ	PE	mouse IgG1	4S.B3	Becton Dickinson	554552
	KLRG1	PerCP-ef710	mouse IgG2a	13F12F2	Thermofisher	46-9488-42
	TCRgd	BV510	mouse IgG1	11F2	Becton Dickinson	745026
	TIGIT	AF647	mouse IgG1	MBSA43	eBioscience	51-9500-42
	TNF-α	AF700	mouse IgG1	Mab11	Biolegend	502928
	XCL1	uncojugated	goat IgG	polyclonal	R&D Systems	AF695
Secondary	Mouse IgG1	AF594	goat IgG	polyclonal	Invitrogen	A-21125
Secondary	Goat IgG	AF488	donkey IgG	polyclonal	Invitrogen	A-11055

C. Antibodies T cell panel sorting

Specificity		Antibody characteristics				
**CD designation**	**Alternative name**	**Fluorochrome**	**Isotype**	**Clone**	**Company**	**Catalog#**
CD3		BV510	mouse IgG1	UCHT1	Biolegend	300448
CD4		PE-Cy5.5	mouse IgG1	13B8.2	Beckman Coulter	B16491
CD8		APC-H7	mouse IgG1	SK1	Becton Dickinson	560179
CD27	TNFRSF7	BV421	mouse IgG1	M-T271	Becton Dickinson	562513
CD28		APC	mouse IgG1	CD28.2	Biolegend	302912
CD31	PECAM-1	PE	mouse IgG1	MEM-05	Exbio	1P-273-T100
CD45RA		PE-TxR	mouse IgG2b	MEM-56	Invitrogen	MHCD45RA17
CD197	CCR7	FITC	mouse IgG2a	150503	R&D Systems	FAB197F-100

Table S2 peptides

	Sequence	Region
1	SKVYLPPTPVSKVISTDVYVTRTNV	L1 HPV2 25-126
2	TDVYVTRTNVYYHGGSSRLLTVGHP	L1 HPV2 25-126
3	SSRLLTVGHPYYSIKKSNNKVAVPK	L1 HPV2 25-126
4	KSNNKVAVPKVSGYQYRVFHVKLPD	L1 HPV2 25-126
5	YRVFHVKLPDPNKFGLPDADLYDPD	L1 HPV2 25-126
6	LPDADLYDPDTQRLLWACVGVEVGR	L1 HPV2 25-126
7	AAEPYGDSMFFSLRREQMFTRHFFN	L1 HPV2 250-351
8	EQMFTRHFFNLGGKMGDTIPDELYI	L1 HPV2 250-351
9	GDTIPDELYIKSTSVPTPGSHVYTS	L1 HPV2 250-351
10	PTPGSHVYTSTPSGSMVSSEQQLFN	L1 HPV2 250-351
11	MVSSEQQLFNKPYWLRRAQGHNNGM	L1 HPV2 250-351
12	RRAQGHNNGMCWGNRVFLTVVDTTR	L1 HPV2 250-351
13	QDTYRYLQSQAITCQKPTPPKTPTD	L1 HPV2 425-449

Table S3 QC metrics TRB sequencing

Sample	raw sequences	useable sequences	productive sequences	clonotypes
UPN1 HPV- T cell cultured	84591	84090	80528	197
UPN1 HPV +/- T cell cultured	93102	92468	64169	250
UPN1 HPV+ T cell cultured	69249	68709	51521	359
UPN1 HPV+ skin	90948	87527	59195	337
UPN1 CD4 memory	99182	98583	87192	3308
UPN1 CD4 naive	97713	97111	79872	7857
UPN1 CD8 memory	94810	94187	71031	1321
UPN1 CD8 naive	90857	90302	72635	7449
HC CD4 memory	102501	102082	91499	3607
HC CD4 naive	79053	78410	64530	8891
HC CD8 memory	77150	76386	48413	984
HC CD8 naive	84738	84183	66778	5578
UPN1 peptide 11	45791	36606	14841	107
UPN1 peptide 12	47715	37858	14704	105

Table S4. Input Vbeta populations per sample

Total

vb	UPN1_1	UPN1_2	UPN1_3	HD	
TCRA_vb3	1282	2440	7720	10323	
TCRA_vb5.3	89	200	761	858	
TCRA_vb7.1	161	448	725	1326	
TCRA_vbneg	24842	106717	144780	124658	
TCRB_vb16	57	291	388	1232	
TCRB_vb17	566	3172	5331	4698	
TCRB_vb9	313	830	1244	2326	
TCRB_vbneg	25439	105512	147024	128911	
TCRC_vb18	168	483	860	344	
TCRC_vb20	88	198	508	2550	
TCRC_vb5.1	815	3053	2992	4056	
TCRC_vbneg	25304	106069	149627	130216	
TCRD_vb13.1	849	2434	2617	3497	
TCRD_vb13.6	258	1327	1870	1885	
TCRD_vb8	856	2746	7046	3970	
TCRD_vbneg	24412	103298	142455	127816	
TCRE_vb12	1324	4040	1304	1979	
TCRE_vb2	2373	6737	6577	10757	
TCRE_vb5.2	91	198	3361	821	
TCRE_vbneg	22585	98830	142743	123610	
TCRF_vb1	374	1168	1902	4656	
TCRF_vb21.3	259	1233	1392	1882	
TCRF_vb23	77	192	496	684	
TCRF_vbneg	25665	107213	150196	129946	
TCRG_vb11	191	393	860	1493	
TCRG_vb14	648	3867	2683	5209	
TCRG_vb22	305	2486	3704	3682	
TCRG_vbneg	25231	103059	146740	126782	
TCRH_vb13.2	305	796	3065	3898	
TCRH_vb4	948	3636	11982	2988	
TCRH_vb7.2	269	871	1300	1961	
TCRH_vbneg	24853	104502	137640	128320	
**Total**	**210997**	**878439**	**1231893**	**1097334**	**3418663**
A	26374	109805	153986	137165	
B	26375	109805	153987	137167	
C	26375	109803	153987	137166	
D	26375	109805	153988	137168	
E	26373	109805	153985	137167	
F	26375	109806	153986	137168	
G	26375	109805	153987	137166	
H	26375	109805	153987	137167	

CD4

vb	UPN1_1	UPN1_2	UPN1_3	HD	
TCRA_vb3	1135	1791	3697	6650	
TCRA_vb5.3	80	172	496	585	
TCRA_vb7.1	85	164	486	742	
TCRA_vbneg	14008	38350	67045	71953	
TCRB_vb16	274	625	1096	1774	
TCRB_vb17	52	206	299	978	
TCRB_vb9	150	360	4130	3555	
TCRB_vbneg	14935	39593	64977	72519	
TCRC_vb18	341	747	1659	3276	
TCRC_vb20	164	465	822	312	
TCRC_vb5.1	67	151	444	1847	
TCRC_vbneg	14818	38628	68933	73760	
TCRD_vb13.1	741	2180	1537	2984	
TCRD_vb13.6	759	1669	2345	2708	
TCRD_vb8	154	424	1517	1450	
TCRD_vbneg	14028	36124	67003	72746	
TCRE_vb12	2097	4817	4620	8010	
TCRE_vb2	87	173	310	644	
TCRE_vb5.2	141	274	665	1172	
TCRE_vbneg	12982	34817	64958	70208	
TCRF_vb1	211	478	1217	2312	
TCRF_vb21.3	214	537	531	1298	
TCRF_vb23	55	108	304	362	
TCRF_vbneg	15164	39824	71112	76212	
TCRG_vb11	134	214	469	676	
TCRG_vb14	131	319	737	2785	
TCRG_vb22	173	487	1015	2172	
TCRG_vbneg	15119	41489	72202	74857	
TCRH_vb13.2	867	3215	11548	2020	
TCRH_vb4	188	318	685	1014	
TCRH_vb7.2	222	430	2296	2467	
TCRH_vbneg	14478	37985	60117	75035	
**Total**	**124054**	**327134**	**579272**	**639083**	**1669543**
A	15308	40477	71724	79930	
B	15411	40784	70502	78826	
C	15390	39991	71858	79195	
D	15682	40397	72402	79888	
E	15307	40081	70553	80034	
F	15644	40947	73164	80184	
G	15557	42509	74423	80490	
H	15755	41948	74646	80536	

CD8 & CD4-CD8-

vb	UPN1_1	UPN1_2	UPN1_3	HD	
TCRA_vb3	147	644	4017	3666	
TCRA_vb5.3	9	27	262	273	
TCRA_vb7.1	68	259	234	579	
TCRA_vbneg	7052	38527	61347	34194	
TCRB_vb16	39	196	143	548	
TCRB_vb17	5	85	89	252	
TCRB_vb9	416	2805	1195	1116	
TCRB_vbneg	6535	33994	65806	37638	
TCRC_vb18	474	2290	1326	768	
TCRC_vb20	4	15	27	29	
TCRC_vb5.1	21	47	63	699	
TCRC_vbneg	6644	37260	63549	37977	
TCRD_vb13.1	112	556	5503	963	
TCRD_vb13.6	88	757	269	786	
TCRD_vb8	104	897	349	421	
TCRD_vbneg	6656	36639	58504	36003	
TCRE_vb12	276	1913	1955	2735	
TCRE_vb2	4	24	3030	176	
TCRE_vb5.2	1183	3766	637	799	
TCRE_vbneg	5381	33221	60293	34146	
TCRF_vb1	162	690	685	2344	
TCRF_vb21.3	44	695	857	582	
TCRF_vb23	22	82	191	321	
TCRF_vbneg	6898	37889	63135	35664	
TCRG_vb11	56	177	386	643	
TCRG_vb14	514	3517	1900	2395	
TCRG_vb22	132	1986	2684	1501	
TCRG_vbneg	6196	30404	57447	33383	
TCRH_vb13.2	76	410	432	962	
TCRH_vb4	81	552	614	941	
TCRH_vb7.2	77	358	749	1376	
TCRH_vbneg	6742	36862	61123	34379	
**Total**	**56218**	**307544**	**518801**	**308259**	**1190822**
A	7276	39457	65860	38712	
B	6995	37080	67233	39554	
C	7143	39612	64965	39473	
D	6960	38849	64625	38173	
E	6844	38924	65915	37856	
F	7126	39356	64868	38911	
G	6898	36084	62417	37922	
H	6976	38182	62918	37658	

A **UPN1**

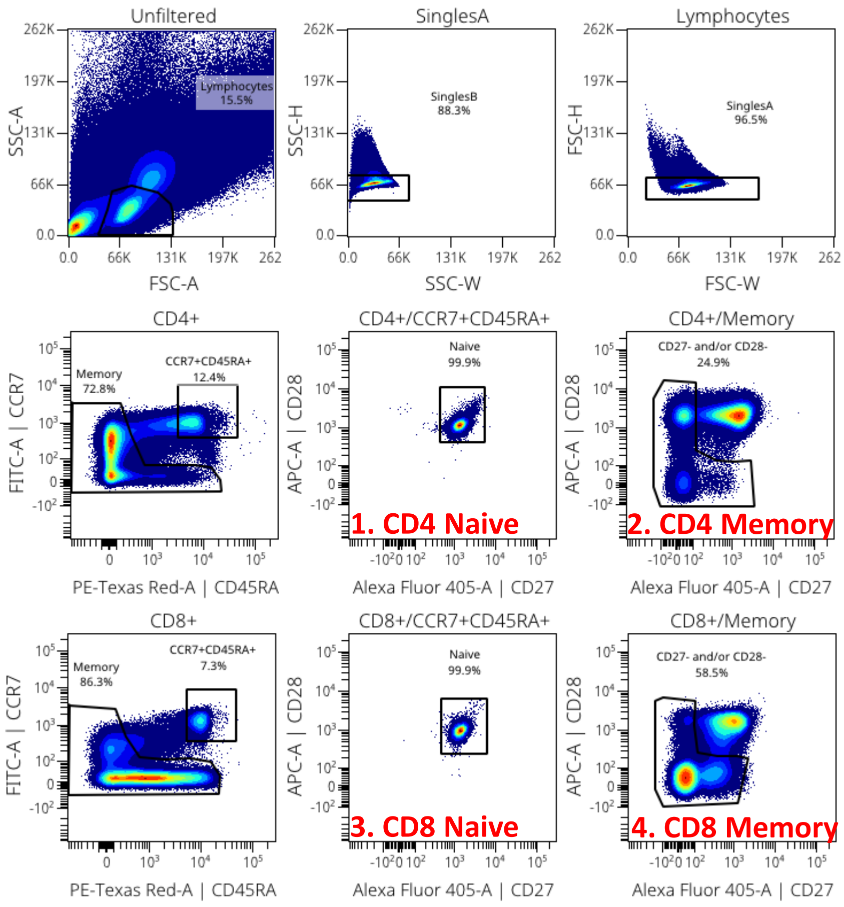

B **Healthy control** **C** **Healthy donor**

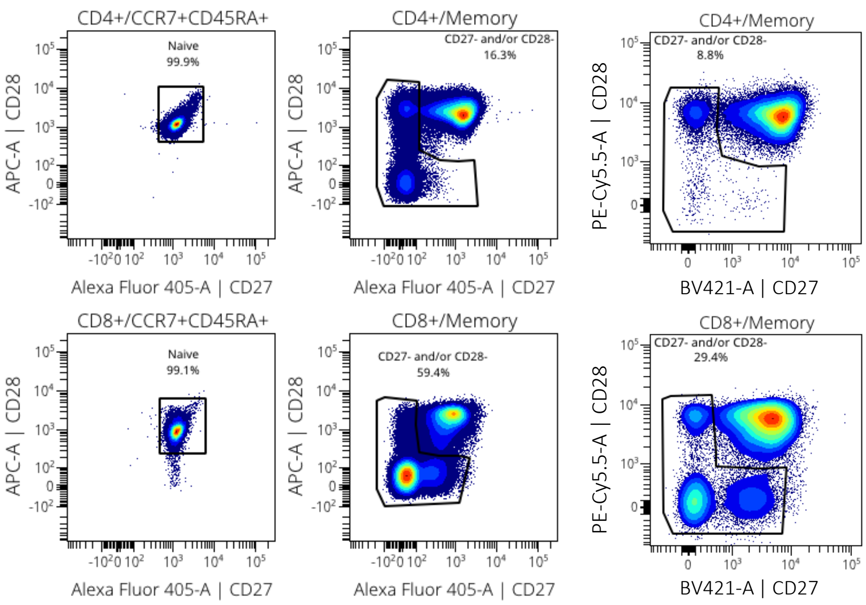

D

	UPN1	Healthy control
1. CD4 Naive	530514	403768
2. CD4 Memory	571955	170211
3. CD8 Naive	107628	83247
4. CD8 Memory	638882	415016

Figure S1. Gating strategy for sorting T cell populations for TCRB sequencing

A) The gating strategy applied to sort the naive and $CD27^-$ and/or $CD28^-$ memory populations of CD4 and CD8 T cells is shown for UPN1. **B)** The sorted fractions of the age-matched healthy control. **C)** In comparison to UPN1 and the healthy control, the percentage of $CD27^-$ and/or $CD28^-$ memory T cells in the healthy donor was much lower. Because of limited available material, no sorting could be performed of a healthy donor sample. **D)** The total number of cells sorted for each individual population per sample.

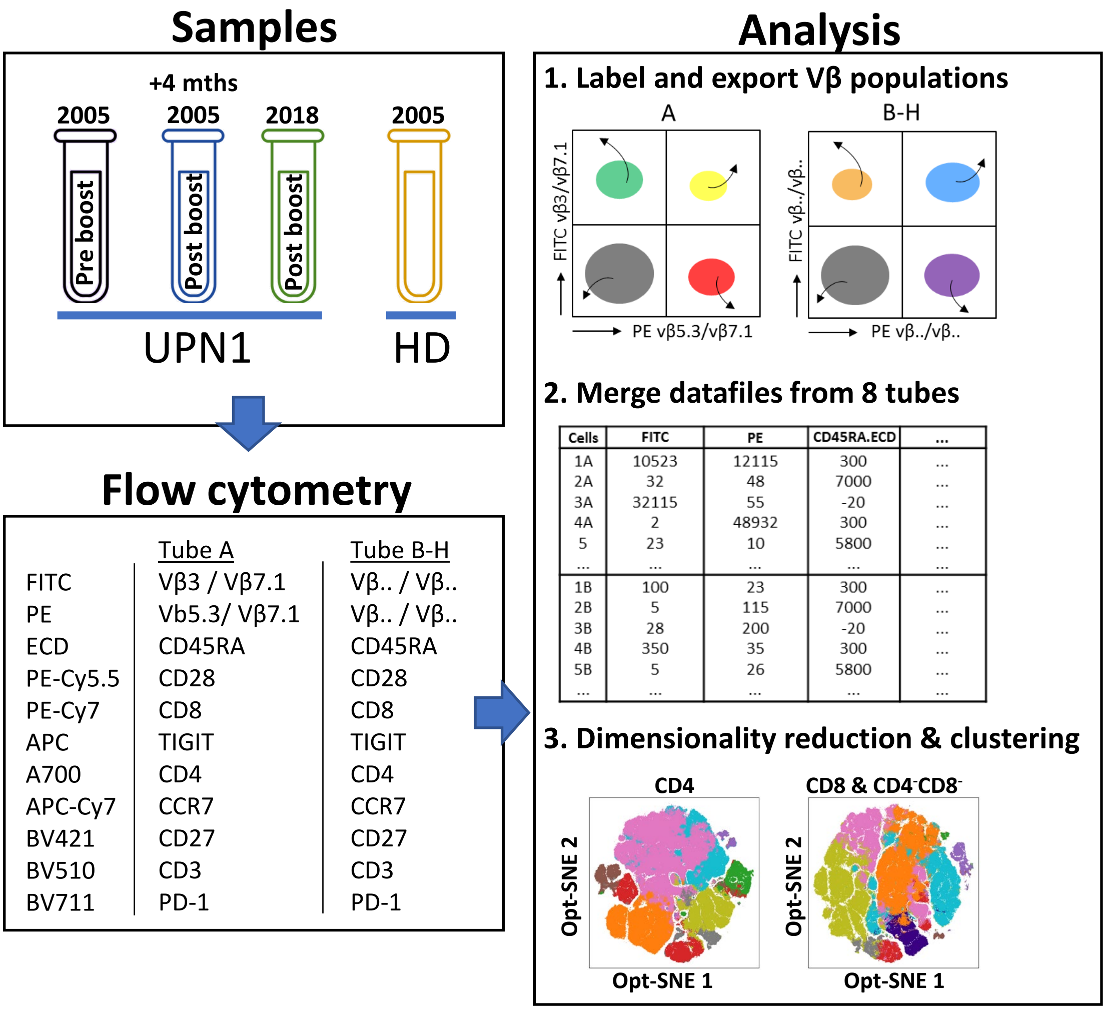

Figure S2. Pipeline T cell receptor Vβ analysis combined with phenotyping

PBMC from UPN1 (pre-boost, 4 months post-boost and 13 years after the boost) and his donor were phenotyped by flow cytometry. The panel consisted of 8 tubes, each including three Vβ antibodies and a backbone panel of lineage, differentiation and activation markers. Analysis was performed by pre-gating each individual Vβ population, after which the data from the 8 tubes were combined. Next, opt-SNE was performed as dimensionality reduction tool on the CD4 and CD8 & $CD4^-CD8^-$ T cells. Clustering was performed by FlowSOM.

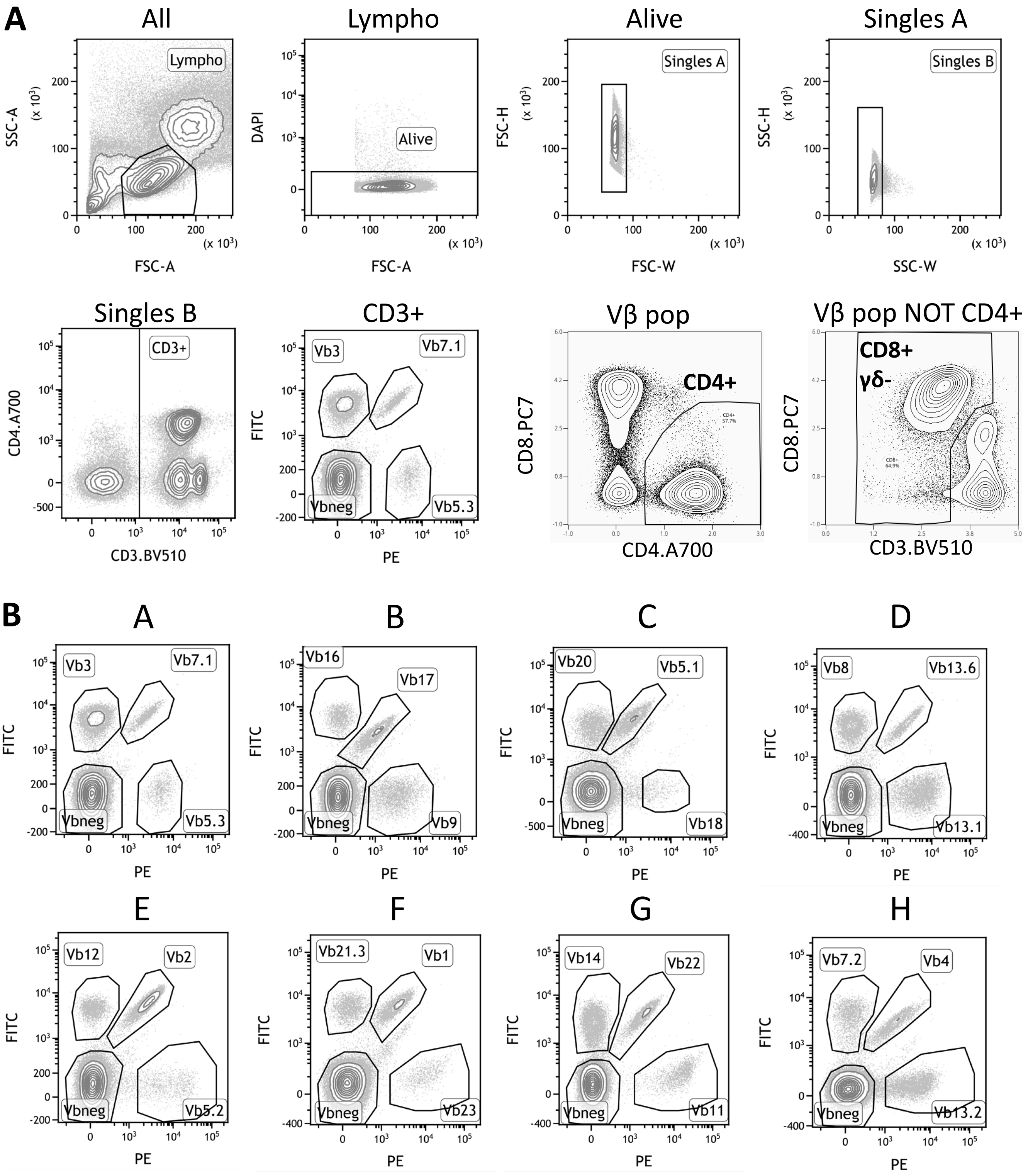

Figure S3. Gating strategy for Vβ populations

A) The gating strategy applied to select T cells includes selection of living single lymphocytes and subsequently T cells based on CD3 expression. The Vβ populations were selected based on FITC and PE expression (in this example tube A) and uploaded on the OMIQ platform. Next, each individual Vβ population was separated into a $CD4^+$ population and $CD4^-CD8^{+/-}$ population. $CD4^-CD3^{high}$ T cells were removed for further analysis since they are likely to represent γδ T cells. **B)** The gating strategy for selection of the Vβ populations for each individual tube is shown.

A

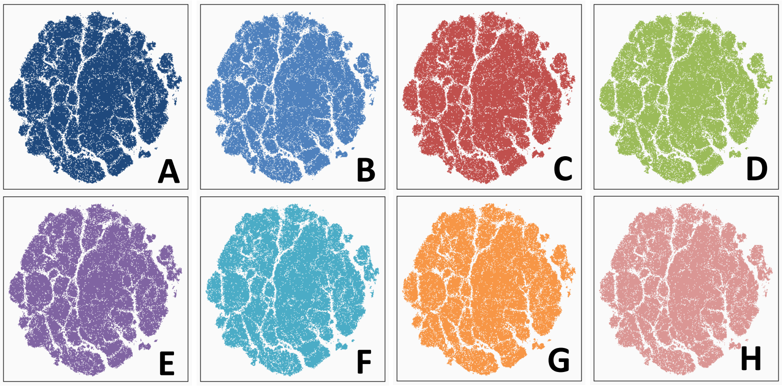

B CD8 T cells

	Cluster 1	Cluster 2	Cluster 3	Cluster 4	Cluster 5	Cluster 6	Cluster 7	Cluster 8	Cluster 9	Cluster 10
TCRA	32.3	0.4	3.79	2.23	0.46	17.8	2.04	22.3	13.9	4.77
TCRB	31.6	0.4	4.19	2.53	0.52	18.1	2.11	22	13.5	5.07
TCRC	31.7	0.42	4.03	2.46	0.48	17.8	2.12	22.4	13.7	4.89
TCRD	32.5	0.37	3.93	2.36	0.46	18.3	2.02	21.7	13.7	4.62
TCRE	31.8	0.43	4.06	2.53	0.45	18.2	2.05	22.6	13.4	4.44
TCRF	31	0.39	3.55	2.24	0.54	17.9	2.05	22.7	13.7	5.84
TCRG	32.2	0.42	4.02	2.48	0.47	17.9	2.13	22	13.6	4.78
TCRH	32.6	0.38	3.8	2.32	0.45	18	2.03	22.3	13.6	4.58

C CD4 T cells

	Cluster 1	Cluster 2	Cluster 3	Cluster 4	Cluster 5	Cluster 6	Cluster 7	Cluster 8	Cluster 9
TCRA	20.00	5.62	8.55	3.45	42.60	2.60	10.00	6.42	0.68
TCRB	19.90	5.23	8.86	3.61	42.40	2.75	10.20	6.30	0.71
TCRC	19.90	5.73	8.75	3.59	42.30	2.73	9.89	6.40	0.69
TCRD	19.30	5.41	8.65	3.66	42.90	2.74	10.10	6.44	0.72
TCRE	19.30	5.67	8.90	3.29	42.80	2.77	10.30	6.35	0.69
TCRF	20.30	5.61	8.44	3.41	42.40	2.32	10.20	6.67	0.68
TCRG	18.90	5.68	8.79	3.66	42.50	2.48	10.20	7.05	0.74
TCRH	19.10	5.48	8.94	3.53	42.60	2.44	10.10	7.09	0.68

Figure S4. Quality control
To preclude the possibility that differences among tubes were driving the clustering we checked whether each tube was equally represented within each cluster. **A)** As an example, the distribution of each tube is projected on the opt-SNE embedding of the CD8 T cells. The percentage of cells within each cluster represented by the different tubes are shown for **B)** the CD8 T cells and **C)** the CD4 T cells.

A

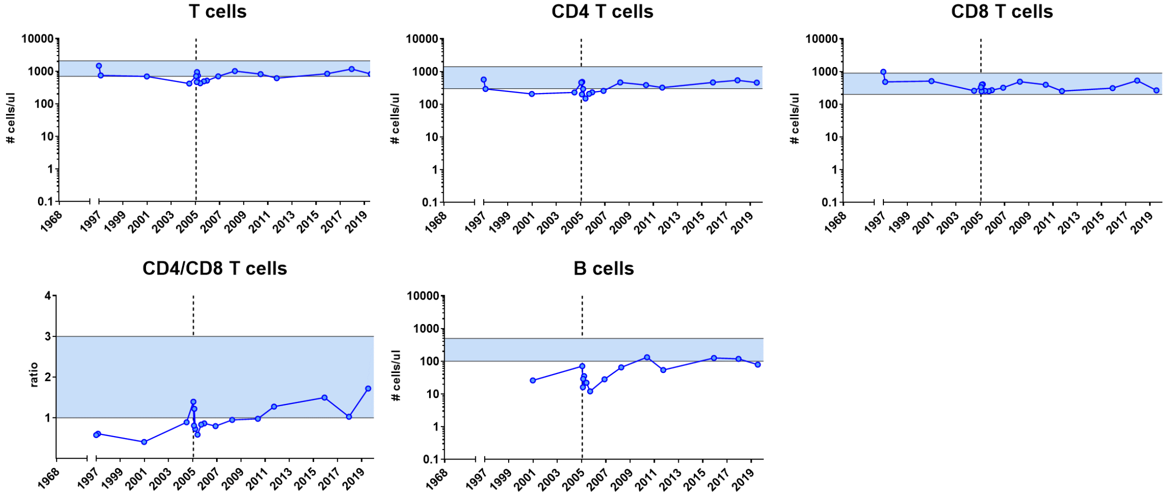

B

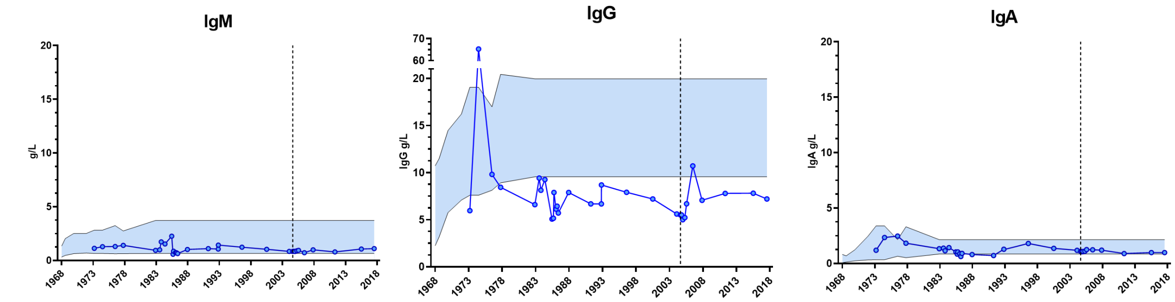

C

	Total B	Naive B	Unswitched memory B	Switched memory B
2001 (pre boost)	0%			
2019 (post boost)		29%	49%	93%

Figure S5. Immunological parameters UPN1

A) Total T, CD4 T, CD8 T and B cells, and **B)** immunoglobulin levels measured as routine diagnostic tool. The dotted line represents the peripheral stem cell boost. **C)** Donor chimerism of B cells. Naive, unswitched memory and switched memory B cells were defined as $CD19^+CD27^-IgD^+IgM^+$, $CD19^+CD27^+IgD^+IgM^+$ and $CD19^+CD27^+IgD^-IgM^-$, respectively.

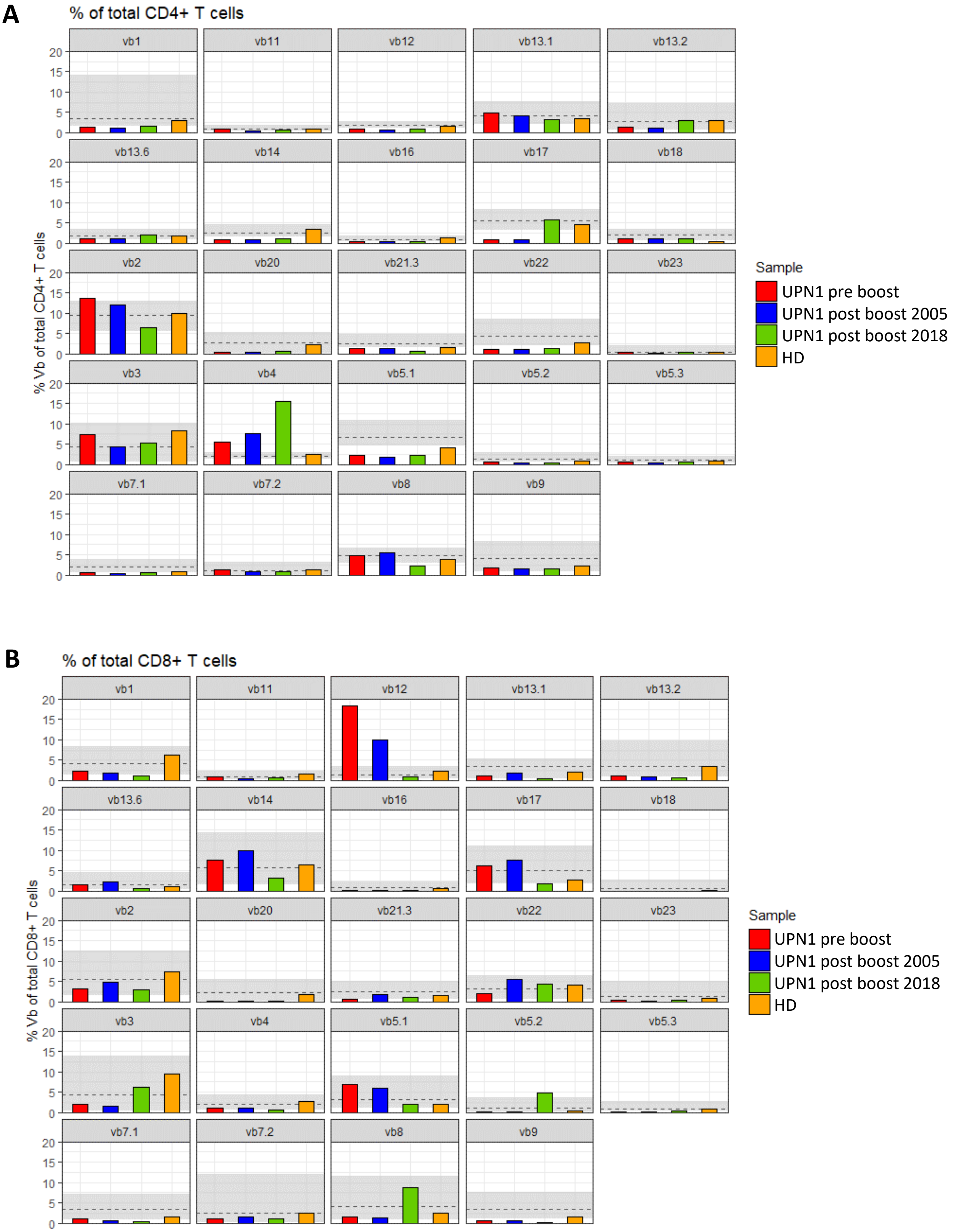

Figure S6. Vβ frequency among total CD4 and CD8 T cells

For each individual sample the Vβ frequency is calculated as percentage of **A)** total CD4 T cells and **B)** total conventional CD8 T cells. The minimum and maximum reference values, measured among at least 46 healthy donors by the manufacturer of the Vβ antibody kit is indicated in grey. The dashed line indicates the mean reference value.

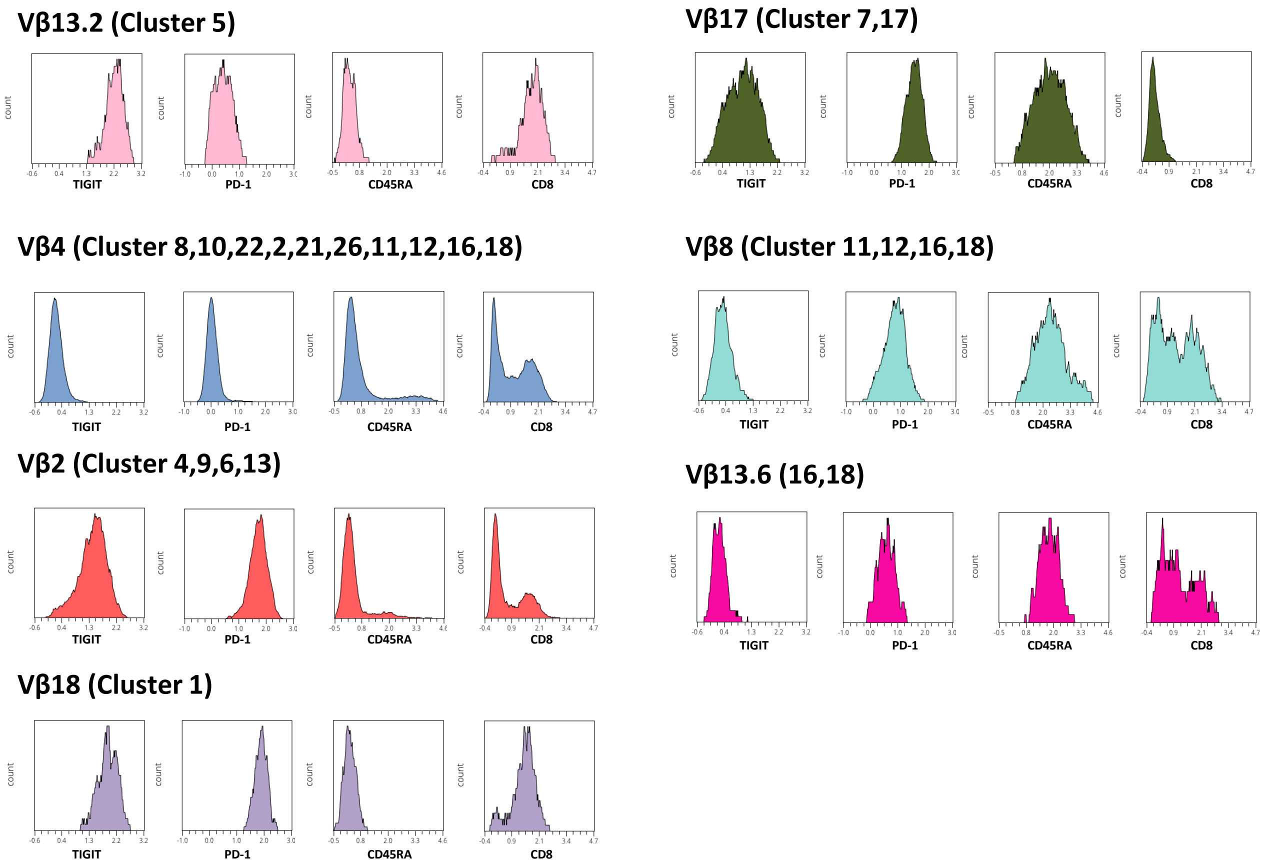

Figure S7. Expression of TIGIT, PD-1, CD45RA and CD8 of enriched Vβ families within CD27⁻ and/or CD28⁻ memory CD4 population

For each dominant Vβ an overlay histogram is shown of the clusters in which the Vβ family was enriched. The four samples (UPN1 pre boost, post boost 2005, post boost 2018 and healthy donor) were pooled.

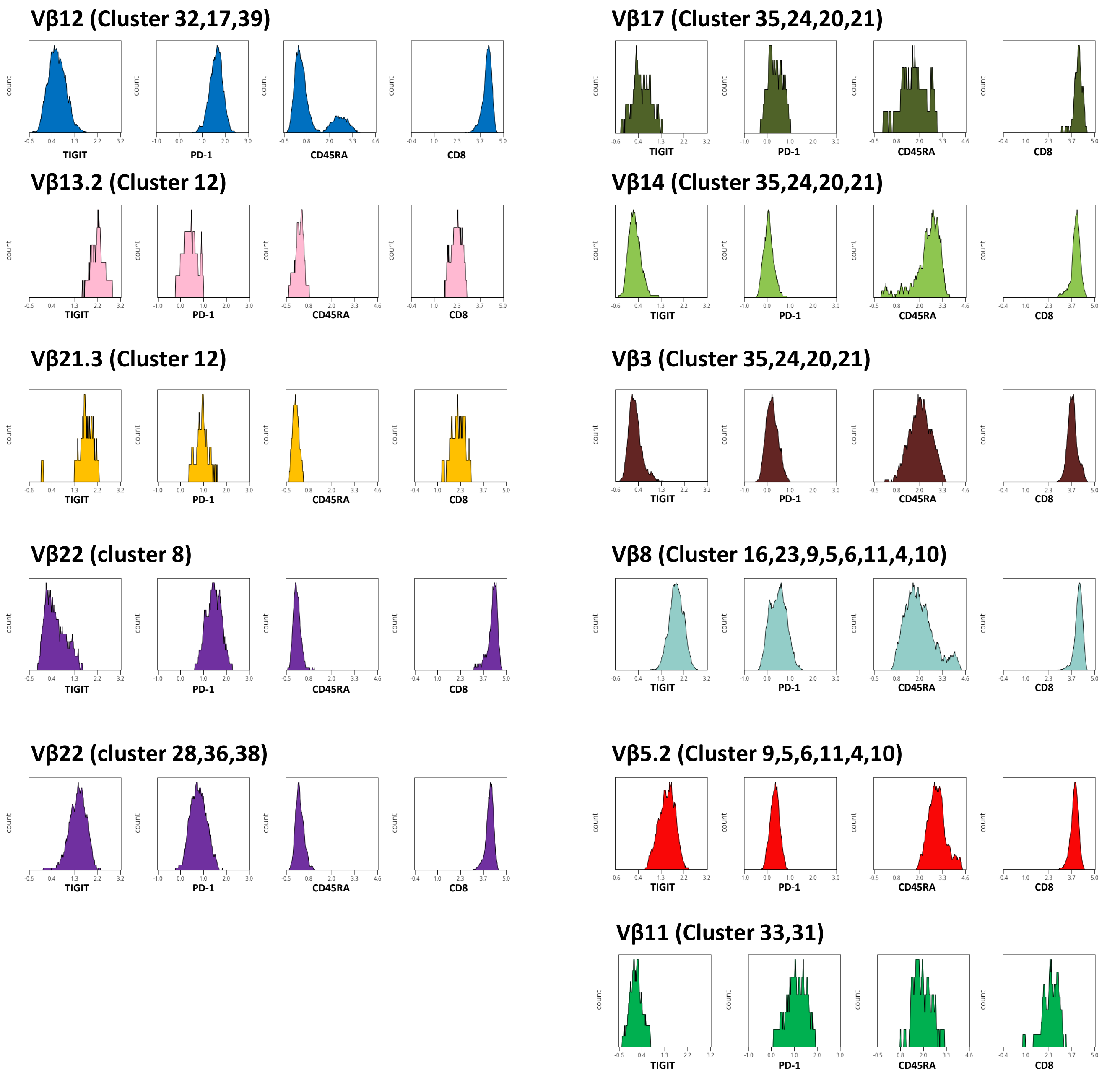

Figure S8. Expression of TIGIT, PD-1, CD45RA and CD8 of enriched Vβ families within $CD27^-$ and/or $CD28^-$ memory CD8 T cell population

For each dominant Vβ an overlay histogram is shown of the clusters in which the Vβ was enriched. The four samples (UPN1 pre boost, post boost 2005, post boost 2018 and healthy donor) were pooled.

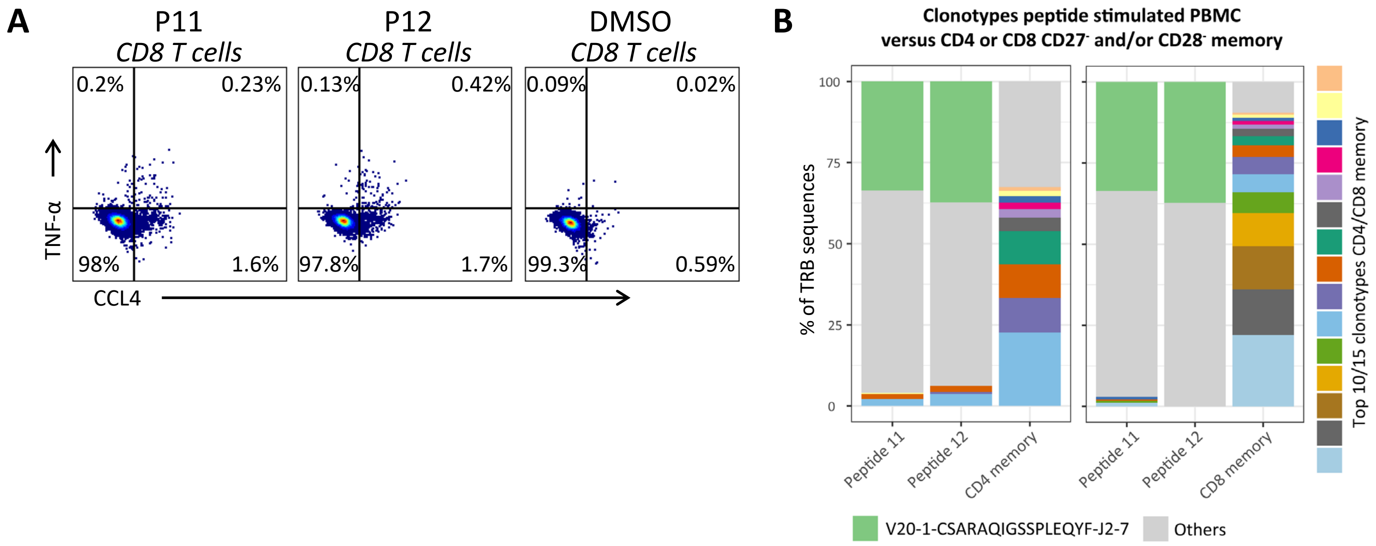

Figure S9. HPV2-derived peptide stimulation of PBMC

A) PBMC of UPN1 were cultured for 7 days with peptide 11 or peptide 12 (Table S2). The TNF-α and CCL4 production of CD8 T cells is shown. **B)** TRB sequencing results of PBMC of UPN1 cultured for 26 days with single peptide. Only clonotypes with at least 50 reads were included. The most frequent clonotype in both peptide cultures is depicted in green. The top 10/15 most frequent clonotypes of the sorted CD4 and CD8 memory populations, as indicated in Figure 7B are colored to show the overlap with the peptide cultured cells.
